# Integrated plasma proteomics identifies tuberculosis-specific diagnostic biomarkers

**DOI:** 10.1172/jci.insight.173273

**Published:** 2024-03-21

**Authors:** Hannah F. Schiff, Naomi F. Walker, Cesar Ugarte-Gil, Marc Tebruegge, Antigoni Manousopoulou, Spiros D. Garbis, Salah Mansour, Pak Ho (Michael) Wong, Gabrielle Rockett, Paolo Piazza, Mahesan Niranjan, Andres F. Vallejo, Christopher H. Woelk, Robert J. Wilkinson, Liku B. Tezera, Diana Garay-Baquero, Paul Elkington

**Affiliations:** 1 NIHR Biomedical Research Centre, School of Clinical and Experimental Sciences, Faculty of Medicine, University of Southampton, Southampton, United Kingdom.; 2 Institute for Life Sciences, Southampton, United Kingdom.; 3 Department of Clinical Sciences, Liverpool School of Tropical Medicine, Liverpool, United Kingdom.; 4 Instituto de Medicina Tropical Alexander von Humboldt, Universidad Peruana Cayetano Heredia, Lima, Peru.; 5 Department of Epidemiology, School of Public and Population Health, University of Texas Medical Branch, Galveston, Texas, USA.; 6 Department of Infection, Immunity & Inflammation, Great Ormond Street Institute of Child Health, University College London, London, United Kingdom.; 7 Department of Paediatrics, Klinik Ottakring, Wiener Gesundheitsverbund, Vienna, Austria.; 8 Department of Paediatrics, The University of Melbourne, Parkville, Australia.; 9 Proteas Bioanalytics, The Lundquist Institute for Biomedical Innovation, Harbor-UCLA Medical Center, Torrance, California, USA.; 10 Centre for Human Genetics, University of Oxford, Oxford, United Kingdom.; 11 Electronics and Computer Sciences, Faculty of Engineering and Physical Sciences, University of Southampton, Southampton, United Kingdom.; 12 Verge Genomics, South San Francisco, California, USA.; 13 Centre for Infectious Diseases Research in Africa, Institute of Infectious Diseases and Molecular Medicine, and; 14 Department of Medicine, University of Cape Town, Observatory, Republic of South Africa.; 15 Department of Infectious Diseases, Imperial College London, London, United Kingdom.; 16 The Francis Crick Institute, London, United Kingdom.

**Keywords:** Infectious disease, Pulmonology, Diagnostics, Proteomics, Tuberculosis

## Abstract

**BACKGROUND:**

Novel biomarkers to identify infectious patients transmitting *Mycobacterium tuberculosis* are urgently needed to control the global tuberculosis (TB) pandemic. We hypothesized that proteins released into the plasma in active pulmonary TB are clinically useful biomarkers to distinguish TB cases from healthy individuals and patients with other respiratory infections.

**METHODS:**

We applied a highly sensitive non-depletion tandem mass spectrometry discovery approach to investigate plasma protein expression in pulmonary TB cases compared to healthy controls in South African and Peruvian cohorts. Bioinformatic analysis using linear modeling and network correlation analyses identified 118 differentially expressed proteins, significant through 3 complementary analytical pipelines. Candidate biomarkers were subsequently analyzed in 2 validation cohorts of differing ethnicity using antibody-based proximity extension assays.

**RESULTS:**

TB-specific host biomarkers were confirmed. A 6-protein diagnostic panel, comprising FETUB, FCGR3B, LRG1, SELL, CD14, and ADA2, differentiated patients with pulmonary TB from healthy controls and patients with other respiratory infections with high sensitivity and specificity in both cohorts.

**CONCLUSION:**

This biomarker panel exceeds the World Health Organization Target Product Profile specificity criteria for a triage test for TB. The new biomarkers have potential for further development as near-patient TB screening assays, thereby helping to close the case-detection gap that fuels the global pandemic.

**FUNDING:**

Medical Research Council (MRC) (MR/R001065/1, MR/S024220/1, MR/P023754/1, and MR/W025728/1); the MRC and the UK Foreign Commonwealth and Development Office; the UK National Institute for Health Research (NIHR); the Wellcome Trust (094000, 203135, and CC2112); Starter Grant for Clinical Lecturers (Academy of Medical Sciences UK); the British Infection Association; the Program for Advanced Research Capacities for AIDS in Peru at Universidad Peruana Cayetano Heredia (D43TW00976301) from the Fogarty International Center at the US NIH; the UK Technology Strategy Board/Innovate UK (101556); the Francis Crick Institute, which receives funding from UKRI-MRC (CC2112); Cancer Research UK (CC2112); and the NIHR Biomedical Research Centre of Imperial College NHS.

## Abstract 2

Integrated proteomic profiling and bioinformatic analyses identifies tuberculosis-associated biomarker panels with potential for population screening.

## Graphical abstract

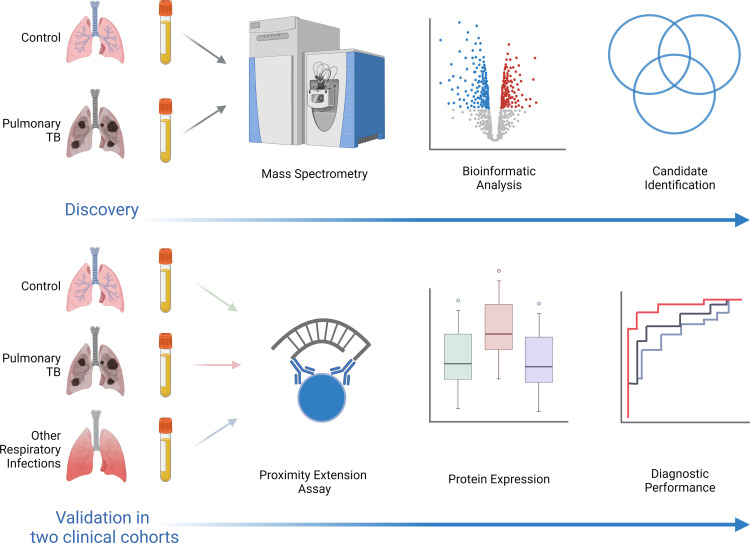

## Introduction

Tuberculosis (TB) remains a disease of global significance, causing 1.3 million deaths and 10.6 million cases of active disease worldwide each year (1). Unfortunately, global control efforts have recently faltered due to the COVID-19 pandemic (2). The World Health Organization (WHO) has identified a global case detection gap of 4 million patients between the estimated incident cases and confirmed diagnoses, with undiagnosed cases predominantly occurring in high-TB-burden countries. Diagnostic delays in low- and middle-income settings are often many months (3) and associate with increased risk of cavitary disease and sputum smear positivity, reflecting high infectiousness (4). Most TB cases result from recently transmitted *Mycobacterium tuberculosis* infection, and therefore the missed diagnoses increase *M*. *tuberculosis* transmission, TB disease and mortality, and fuel the ongoing pandemic (5).

TB control strategies are limited by the currently available diagnostics, which demonstrably are not meeting the needs for global control, requiring specific infrastructure and skilled operators, and do not meet the requirements of the WHO Target Product Profile (TPP) (6). Diagnostic biomarkers capable of identifying people with infectious TB in high-burden settings, ideally at the point of care and not requiring sputum expectoration, are urgently needed. A new screening test would not only benefit individuals by enabling prompt and effective treatment, but would also be a fundamental tool for potential TB elimination, which remains a key goal for the WHO (7).

Proteins are excellent candidates for diagnostic biomarkers, being stable and utilizable for near-patient diagnostic tests. Several studies have explored potential host plasma protein biomarkers of TB (8–16), and although numerous candidate proteins have been detected, biomarkers or combinatorial biomarker signatures have not yet been found that can reliably differentiate TB from other respiratory diseases, or predict progression (17). Most discovery mass spectrometry–based (MS-based) proteomic studies to date have depleted highly abundant protein components from plasma (10–12). This reduction in plasma protein complexity simplifies the analysis, but will also concurrently deplete proteins of biological interest (18–20). Candidate host proteins identified to date as biomarkers of TB disease are frequently highly sensitive but poorly specific (13–15).

We hypothesized that analysis of plasma from individuals with pulmonary TB and healthy controls (HCs) using a non-depletion untargeted proteomics method previously optimized to provide a uniquely high proteome coverage would identify novel markers that achieve both high sensitivity and specificity for TB disease. Here, we report the most detailed plasma proteome of TB to date and perform validation of upregulated proteins by a complementary antibody capture technique in 2 separate clinical cohorts, including patients with other respiratory infections (ORI). We demonstrate the diagnostic potential of an optimized panel incorporating the newly identified biomarkers alongside established analytes that has potential to be developed into a near-patient screening test.

## Results

### Discovery proteomic analysis of non-depleted plasma.

**Figure 1:**
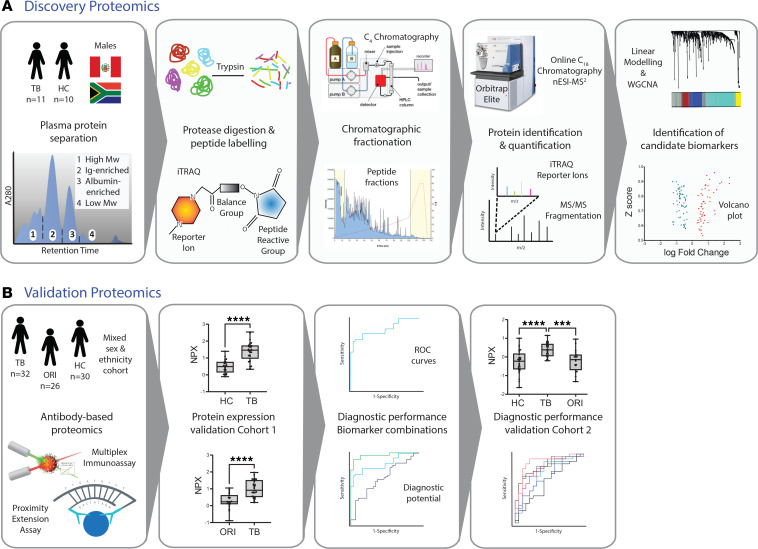
Integrated proteomic study design for TB biomarker identification and validation. (**A**) Discovery stage comprising sequential orthogonal fractionation of non-depleted plasma at both the protein and peptide level, iTRAQ peptide labeling, and tandem mass spectrometry for protein identification and relative quantification. Complementary bioinformatic analysis approaches (linear modeling, using limma, and WGCNA) were then used to identify and prioritize diagnostic biomarkers by combining outputs of these pipelines. (**B**) Candidate protein biomarkers were then validated by multiplex antibody-based techniques (Luminex and proximity extension assay) in serum samples from a separate patient cohort of HCs, pulmonary TB, and ORI of mixed sex and ethnicity. High-performing combinatorial panels were identified for key clinical comparisons and diagnostic performance assessed in 2 separate patient cohorts using binary logistic regression and receiver operating characteristic curves. iTRAQ, isobaric tags for relative and absolute quantification; nESI-MS2, nano-electrospray ionization tandem mass spectrometry; limma, linear modeling for microarray data; WGCNA, whole-gene correlation network analysis; PEA, proximity extension assay; NPX, normalized protein expression; TB, tuberculosis; HC, healthy control; ORI, other respiratory infections; ROC, receiver operating characteristic.

**Figure 2:**
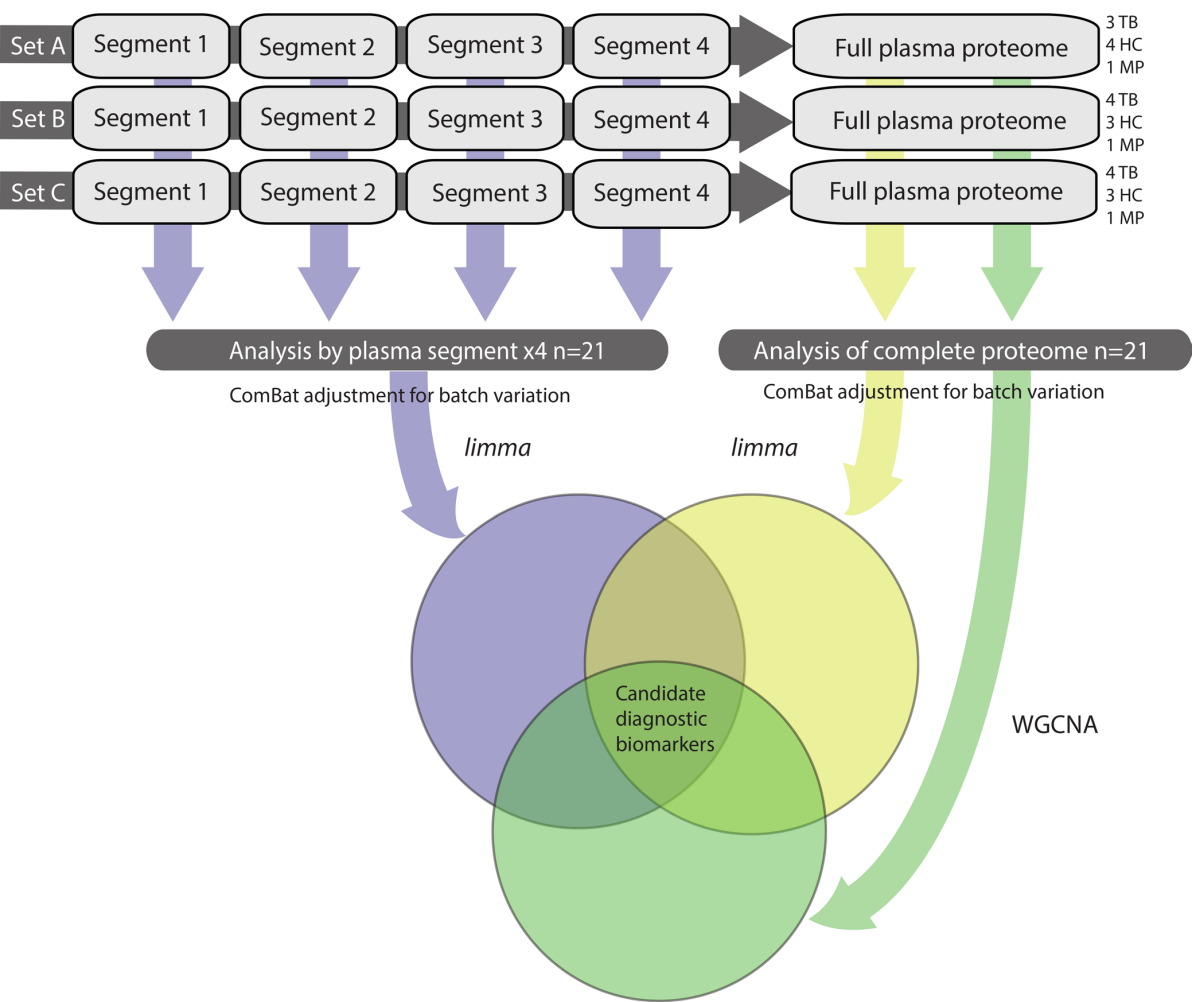
Bioinformatic analysis pipeline. Discovery proteomics experiments were conducted in 12 separate iTRAQ-labeled 8-plex experiments with block randomization of HC and TB samples into 3 experimental sets. Each plasma segment 8-plex experiment included 1 aliquot of a plasma master pool. Grouped protein abundances were calculated across plasma segments for each experimental set to permit analysis over the whole plasma proteome. Protein abundances were then combined by plasma segment and by experimental set and adjusted for experimental batch variation using ComBat. Differential protein expression was analyzed by limma. In parallel, the complete proteome was analyzed by WGCNA to identify protein networks most strongly correlated with TB. Proteins identified as significant by all 3 bioinformatic approaches were then prioritized for validation. iTRAQ, isobaric tags for relative and absolute quantification; ComBat, adjustment for batch effects using an empirical Bayes framework (R package); WGCNA, whole-gene network correlation analysis; limma, linear modeling for microarray data (R package).

The overall study design is presented in Figure 1. Plasma samples were analyzed from 11 untreated male patients with active pulmonary TB and 10 male HC samples, from South African and Peruvian cohorts, using a protocol that involved no depletion steps (21). Each plasma sample was initially separated into 4 segments by size exclusion chromatography, and each segment was processed individually. Analyses of plasma segments were performed in 12 iTRAQ (isobaric tags for relative and absolute quantification) 8-plex experimental sets in a block-randomized design comprising 3 experimental sets. Each iTRAQ experiment contained a bridging master-pool plasma sample run in every experiment. HCs were matched to TB samples by age, ethnicity, and smoking status within each iTRAQ set (Supplemental Tables 1 and 2; supplemental material available online with this article; https://doi.org/10.1172/jci.insight.173273DS1). Protein abundances from the plasma segments and multi-consensus reports were combined and adjusted for experimental batch effects (Supplemental Figures 1 and 2). Protein abundances from 1 TB sample failed normalization, leading to exclusion from downstream analysis. An additional TB sample clustered with controls. On review of the clinical data, the patient had minimal chest x-ray infiltration and normal C-reactive protein (CRP), and so did not fulfil study inclusion criteria, and was also excluded from downstream analysis. Protein abundances from the remaining combined plasma segment proteomes between experimental sets and the combined multi-consensus proteomes were analyzed by complementary bioinformatic approaches to identify candidate diagnostic protein biomarkers (Figure 2). In total, 4,696 protein identifications were made across all iTRAQ experiments, at 5% false discovery rate (FDR). This comprised 2,332 unique host-derived proteins and 22 *M*. *tuberculosis*–derived proteins (Supplemental Table 3). Of these, 594 host proteins had a quantification result for every sample analyzed and therefore comprised the complete quantified proteome. While *M*. *tuberculosis* proteins were identified across all plasma segments, they were identified in both control and disease samples with low confidence and were not analyzed further after review of individual mass spectra.

### Plasma proteomes cluster by clinical condition and geographical cohort.

**Figure 3:**
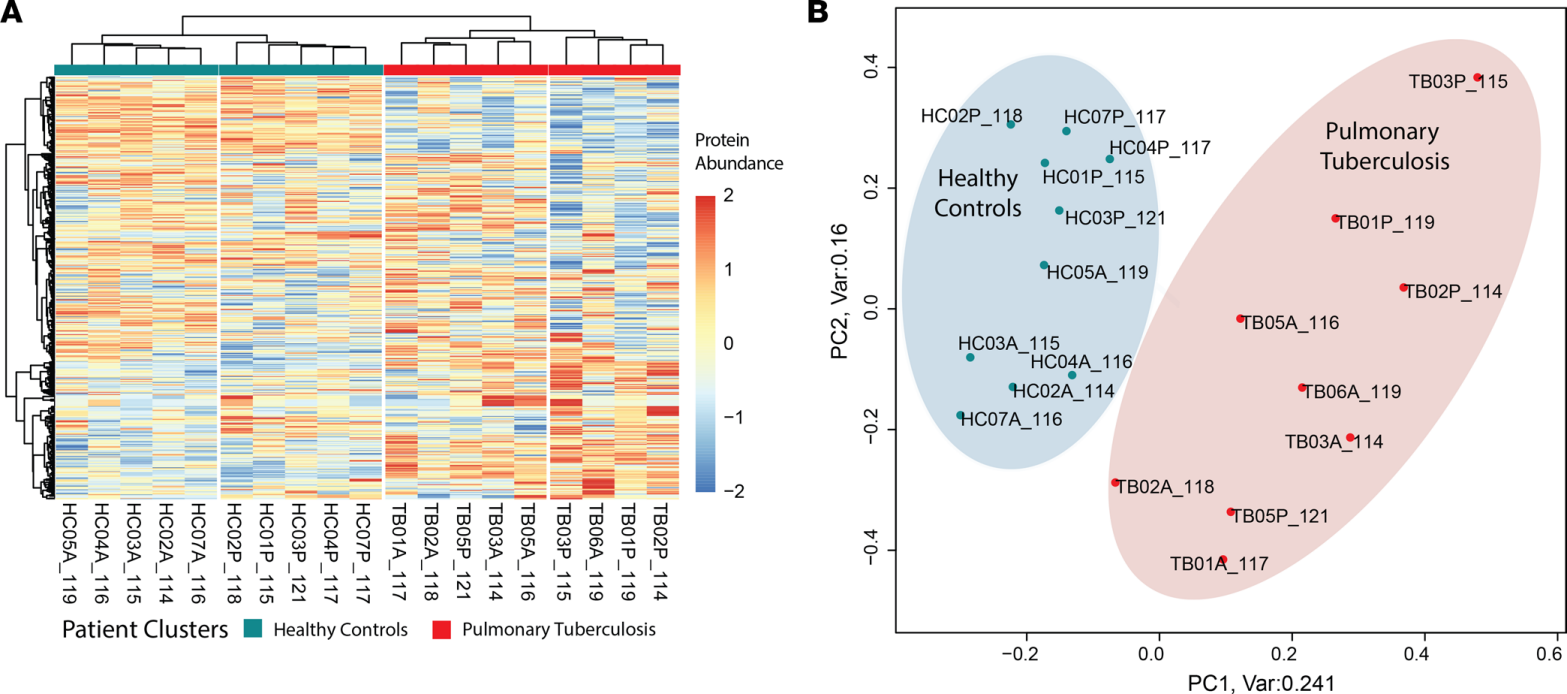
Summary data overview by unsupervised analysis. (**A**) Clustered heatmap for log_2_-transformed fully quantified protein abundances (*n* = 594) shows clear separation of protein abundances between the HC and pulmonary TB groups. iTRAQ tags and clinical groups are indicated. Within HCs, distinct clustering was observed for discovery cohorts of different ethnicity (sample identification: A = South African, P = Peru). This was also observed within the TB group, although some overlap occurred. (**B**) Principal component analysis (PCA) of log_2_-transformed protein abundances demonstrates clear separation by clinical group, responsible for 24% of the variance within the data set. HC, healthy control; iTRAQ, isobaric tags for relative and absolute quantification; TB, tuberculosis.

Initial exploratory data analysis of the complete quantified proteome by unsupervised hierarchical clustering demonstrated clear separation of the clinical groups (Figure 3A). Furthermore, the South African (label A_) and Peruvian cohorts (label P_) separated within clinical groups. This distinction was most marked within the HC plasma samples, with complete segregation depending on geographical location, whereas greater admixture occurred within the TB samples. Similarly, principal component analysis (PCA) confirmed clear separation between clinical groups, manifest by PC1 and comprising 24% of the variation within the data set (Figure 3B). Again, sample clustering by geographical cohort within clinical groups occurred, manifest through PC2, which contained 16% of the variation within the data set (Figure 3B).

### Complementary bioinformatic analysis identifies 118 differentially expressed proteins in pulmonary TB.

**Figure 4:**
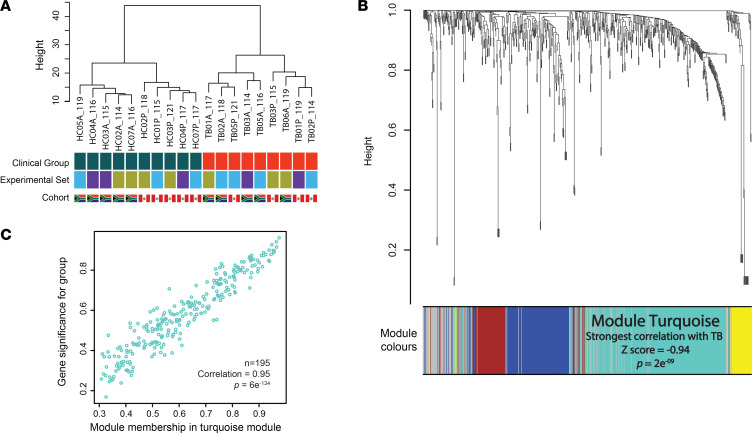
Whole-genome correlation network analysis (WGCNA). (**A**) Hierarchical clustering of samples showing discrete clusters by clinical group and absence of clustering by experimental batch. Discrete clustering by cohort ethnicity is again observed in the HC group, but not in TB patients. (**B**) Protein dendrogram and module colors. Module turquoise, containing 195 proteins, had the strongest correlation with TB (correlation [*z*] score –0.94, *P* = 2 × 10^9^). (**C**) A scatterplot of protein significance by clinical group confirming very high correlation of module turquoise with clinical group (0.95, *P* = 6 × 10^–134^). HC, healthy control; TB, tuberculosis.

High confidence protein identifications, extracted at 1% FDR, were taken forward for differential expression analysis. Protein abundances from individual iTRAQ 8-plex experiments were combined following adjustment for experimental batch (22). FDR-corrected linear modeling (23) identified 195 differentially expressed proteins from analysis of each plasma segment (Supplemental Table 4). A similar *limma* approach analyzing the complete multi-consensus proteome yielded 148 differentially expressed proteins (Supplemental Table 5). In parallel, examining the data set by network correlation methodology (whole-gene correlation network analysis, WGCNA) (24), demonstrated hierarchical clustering by clinical group, but not experimental set, and by cohort in the HCs (Figure 4A). Dendrogram analysis identified a large module of 195 proteins that correlated very closely with disease status (correlation score 0.94, *P* = 2 × 10^–9^) (Figure 4B and Supplemental Table 6). Protein module significance scores within the turquoise module closely correlated to protein significance for pulmonary TB (Figure 4C; *P* = 6 × 10^–134^).

**Figure 5:**
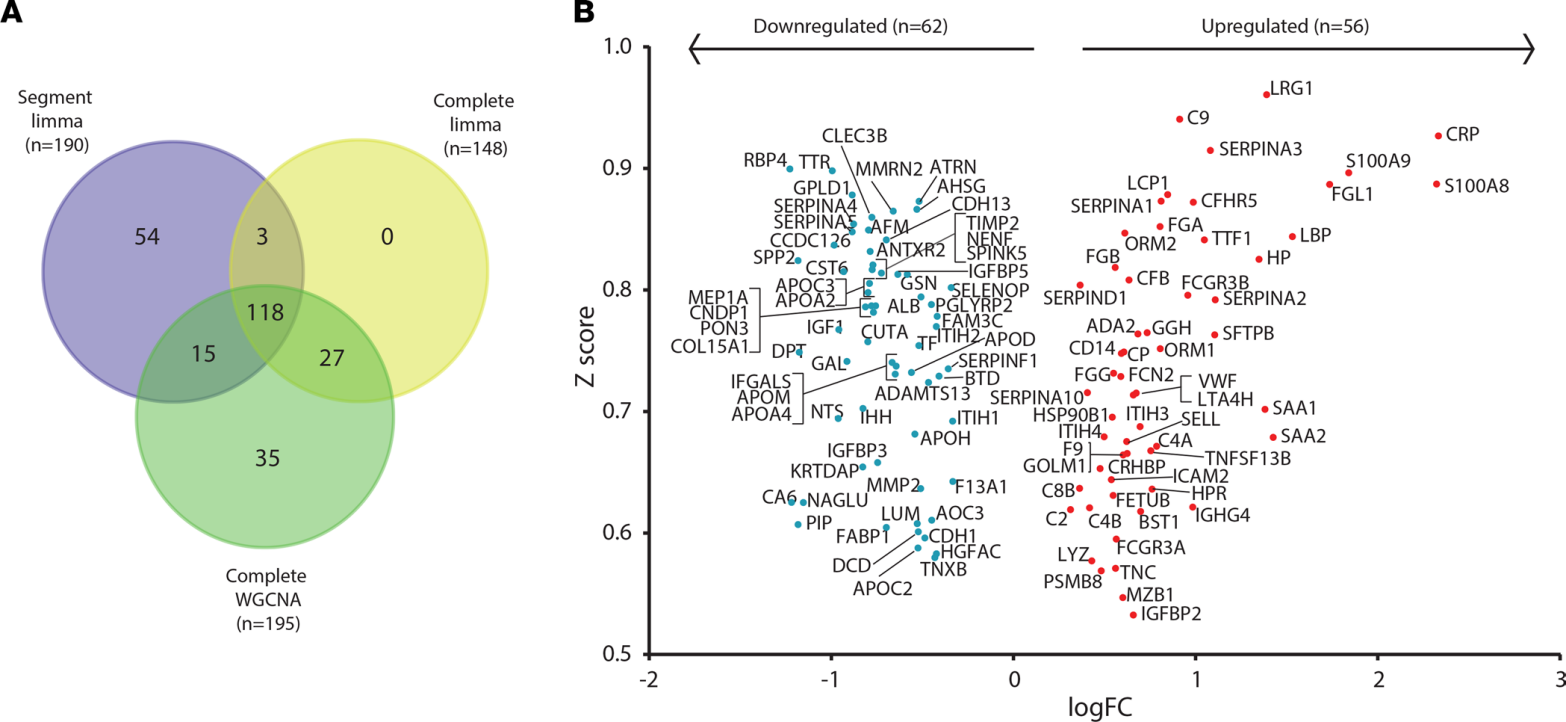
Complementary bioinformatic analyses identify 118 significantly differentially expressed plasma proteins in TB. (**A**) Proteins identified by each bioinformatic approach: 190 from *limma* analysis of segmental plasma proteomes, 148 by *limma* analysis of complete plasma proteomes, and 195 proteins within WGCNA module turquoise. One hundred and eighteen proteins were found to be significantly differentially expressed via all 3 analytical approaches. (**B**) Volcano plot of all 118 significantly differentially expressed proteins by log_2_(fold change) by *limma* and correlation (*z*) score from WGCNA. Markers in the upper outer quadrants have the highest fold changes and strongest correlation to TB. All markers have a *P* value of less than 0.05 after adjustment for multiple testing within *limma*. *limma*, linear modeling for microarray data (R package); WGCNA, whole-genome correlation network analysis.

Combined analysis of all 3 bioinformatic analysis approaches identified 118 proteins that were significant through all statistical approaches (Figure 5A and Supplemental Table 7). Consequently, this group was taken forward as robust candidate diagnostic protein biomarkers. Analysis of protein fold change by *limma* and correlation score by WGCNA demonstrated that 56 proteins were significantly upregulated and 62 were significantly downregulated (Figure 5B).

### Differentially expressed proteins reflect physiological changes in pulmonary TB.

**Figure 6:**
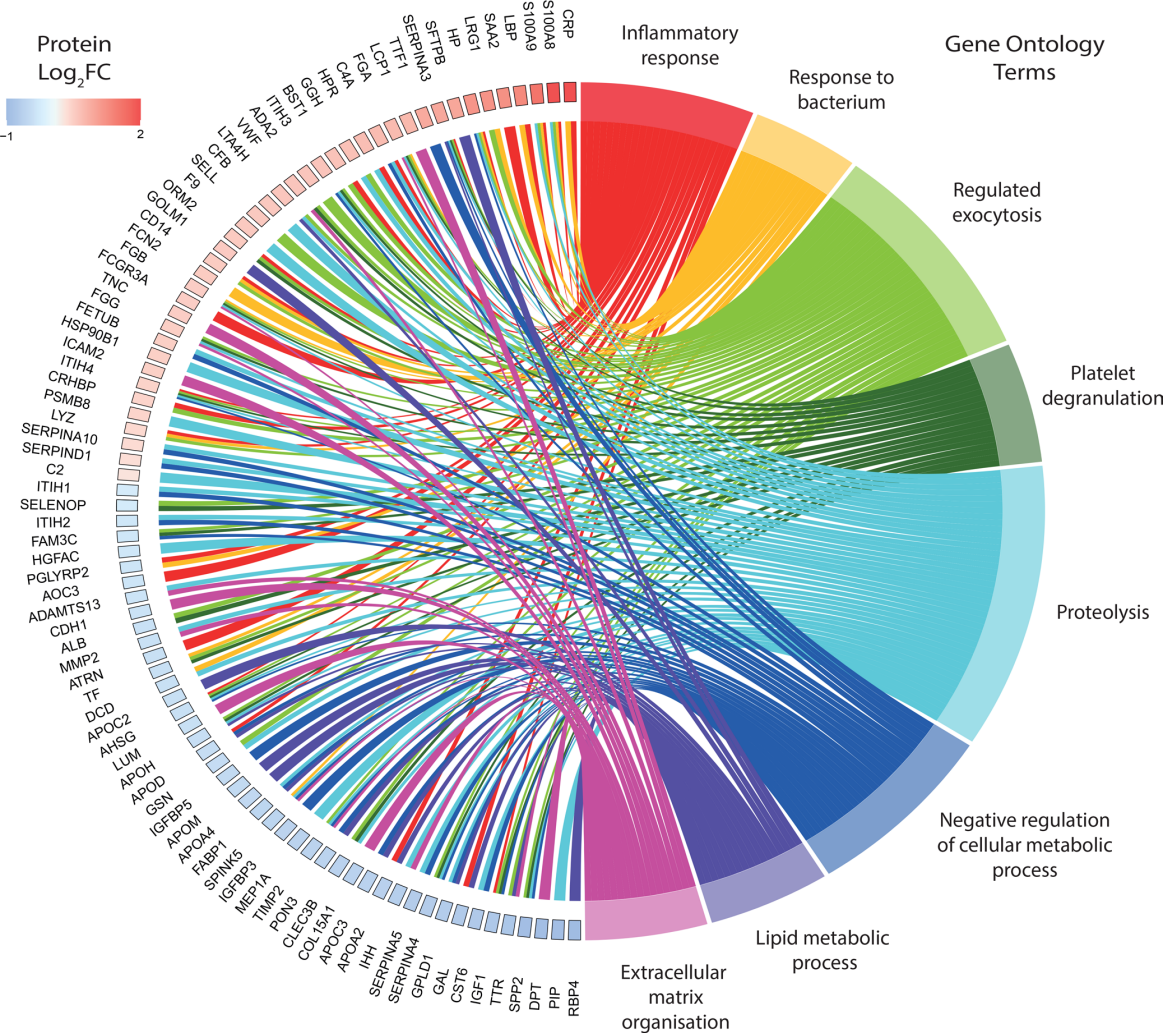
Divergently regulated proteins link with key biological processes in pulmonary TB. A chord plot depicting proteins with a log_2_(fold change) greater than ±0.5 and their links to significantly enriched biological processes in TB. Gene ontology enrichment for biological process was performed using ShinyGO and only significant terms (FDR *q* ≤ 0.05) are shown. Plot generated with the R package GOplots.

**Figure 7:**
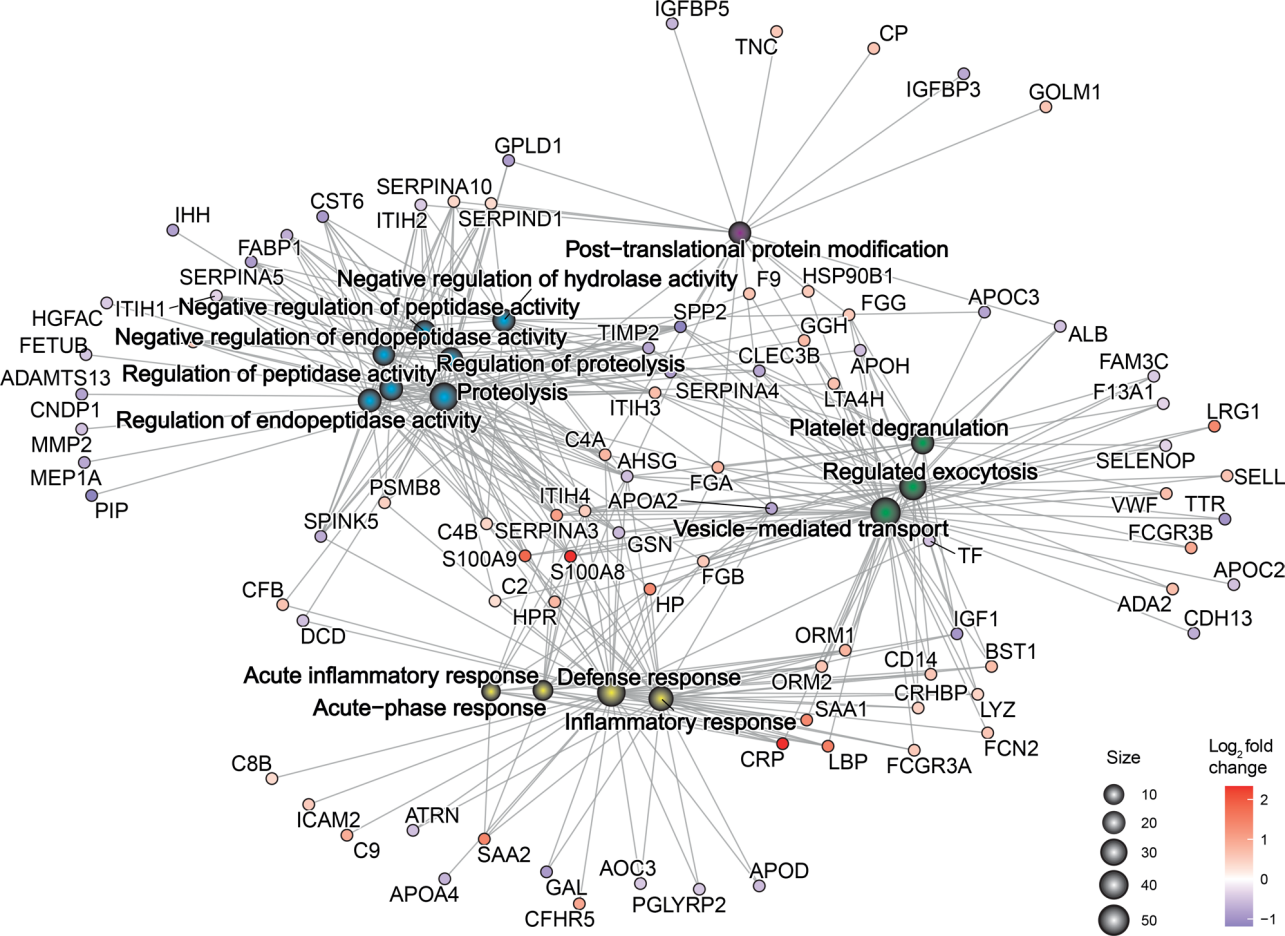
Physiological changes in TB are reflected in the plasma proteome. Functional enrichment analysis by biological process was performed on the 118 differentially expressed plasma proteins in TB. The gene concept network plot depicts the top 15 most enriched biological processes and their links to divergently regulated proteins. Gene ontology enrichment was performed using ShinyGO and the plot was generated using the cnetplot function in the R package GOplots.

Chord plot analysis was performed to demonstrate key proteins and their magnitude and directionality of fold change relative to key biological processes from gene ontology analysis (Figure 6 and Supplemental Table 8). The predominant pathways were consistent with the known biology of *M*. *tuberculosis* infection, such as inflammatory response, response to bacterium, and regulated exocytosis. However, the most represented process was proteolysis, and proteins regulating extracellular matrix organization were also frequent. The final processes were negative regulation of cellular metabolic process, lipid metabolic process, and platelet degranulation. Key proteins related to proteolysis included MMP2, TIMP2, fetuin-B (FETUB), SERPINA3, SERPINA4, SERPINA5, SERPIND1, and SERPINA10. MMP2 and TIMP2 are also key proteins related to extracellular matrix organization, along with the collagen subunit COL15A1, von Willebrand factor (vWF), and ADAMTS13. Proteins related to exocytosis included SELL, CLEC3B, and LTA4H. CRP, lipopolysaccharide-binding protein (LBP), S100A8, and S100A9 were expectedly linked to the acute inflammatory response. LRG1 and CD14 were key proteins in the response to bacterium. Network plot analysis further confirmed the importance of proteolysis, inflammation, and exocytosis-related terms and their relationship to the differentially expressed proteins (Figure 7). Gene ontology analysis of all differentially expressed proteins by cellular compartment showed that the proteins were associated with 6 main locations: endoplasmic reticulum lumen, the extracellular matrix, lipoprotein particles, insulin-like growth factor ternary complexes, secretory vesicles, and platelet granules (Supplemental Table 9). Analysis of enriched molecular function terms indicates significant peptidase and endopeptidase activity, supporting a key role for proteolysis in pulmonary TB (Supplemental Table 10).

Gene ontology analysis of upregulated proteins by cellular component revealed significant enrichment for blood microparticles and fibrinogen complexes (Supplemental Figure 3A), with terms denoting binding to lipid mediators of inflammation and lipopeptides being the dominant molecular functions (Supplemental Figure 3B). Analysis by biological process showed significant enrichment for the acute-phase response and acute inflammatory response (Supplemental Figure 3C and Supplemental Figure 4). The complement and coagulation pathway was the only enriched KEGG pathway by this analysis approach (Supplemental Figure 3D and Supplemental Figure 4). Gene ontology analysis of downregulated proteins was strikingly dominated by lipid-related terms across all analyses (Supplemental Figures 5 and 6).

Proteins forming the matrisome, a group of approximately 1000 genes encoding structural and regulatory proteins of the extracellular matrix (25), were overrepresented within significantly differentiated proteins. Forty-five of the 118 (38%) divergently regulated proteins were from the matrisome, compared with the matrisome representing 5% of the human proteome (26) reflective of increased extracellular matrix turnover in TB (27) (Supplemental Figure 7).

### Proximity extension analysis validates differential protein expression in the plasma of individuals with pulmonary TB in an independent patient cohort.

**Figure 8:**
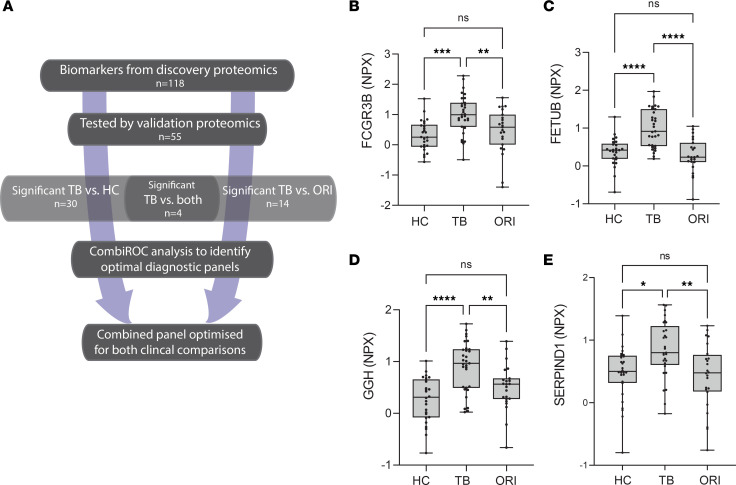
Discovery biomarker candidates validated by proximity extension analysis identify TB-specific biomarkers. (**A**) Flow chart outlining the analysis approach to identify significant biomarkers and the best-performing biomarker combinations from our integrated proteomics approach. (**B**–**E**) Box-and-whisker plots of 4 protein biomarkers significantly differentially expressed in TB compared with both HCs and ORI by proximity extension assay. Boxes show median values and interquartile ranges and whiskers show minimum to maximum values. Statistical differences were calculated using 1-way ANOVA with Tukey’s multiple-comparison test for data with a Gaussian distribution and Kruskal-Willis test with Dunn’s multiple-comparison test for nonparametrically distributed data. NPX, normalized protein expression (log_2_ scale); AUC, area under the curve; HC, healthy control (*n* = 30); TB, tuberculosis; (*n* = 32); ORI, other respiratory infections (*n* = 26); FCGR3B, low-affinity immunoglobulin receptor 3B; FETUB, fetuin-B; GGH, γ-glutamyl hydrolase; SERPIND1, serpin D1, also known as heparin cofactor 2. NS, *P* > 0.05; *P ≤ 0.05; **P ≤ 0.01, ***P ≤ 0.001; ****P ≤ 0.0001.

We performed analysis by an antibody capture–based protein identification approach in an entirely different cohort, studying serum to validate the potential of the MS-identified plasma biomarkers for a new diagnostic panel (Figure 8A). Circulating levels of 55 of the 118 (47%) differentially expressed proteins were tested in an independent patient cohort of mixed ethnicity and sex using an antibody-based proximity extension assay (PEA) (Olink Explore), using cardiometabolic and inflammatory panels, which gave the largest overlap with the 118 differentially expressed proteins. PEA plates hold a maximum of 88 samples, and so to maintain power, 3 groups were analyzed: HC, TB, and ORI. Serum samples were selected from the United Kingdom–based (UK-based) MIMIC cohort (Supplemental Table 11) and included individuals with pulmonary TB (TB, *n* = 32), HCs (*n* = 30) without risk factors for TB infection in whom latent TB infection had been ruled out by a negative IFN-γ release assay, and patients with symptoms suggestive of TB but with microbiologically confirmed ORI (*n* = 26) (Supplemental Table 12). Thirty proteins (30/55, 55%) had confirmed differential expression between HCs and pulmonary TB, of which 25 were upregulated and 5 downregulated (Supplemental Table 13). Fourteen proteins (14/55, 25%) showed differential expression between pulmonary TB and ORI. Four proteins, FCGR3B, FETUB, GGH, and SERPIND1, were present at significantly higher levels in the serum of pulmonary TB patients than both HCs and ORI cases, thereby exhibiting a high degree of specificity for TB (Figure 8B). Significantly reduced circulating levels of RBP4 were demonstrated using Luminex methodology, confirming the findings observed by MS (Supplemental Figure 8).

### A 5-protein panel differentiates pulmonary TB from HCs.

**Figure 9:**
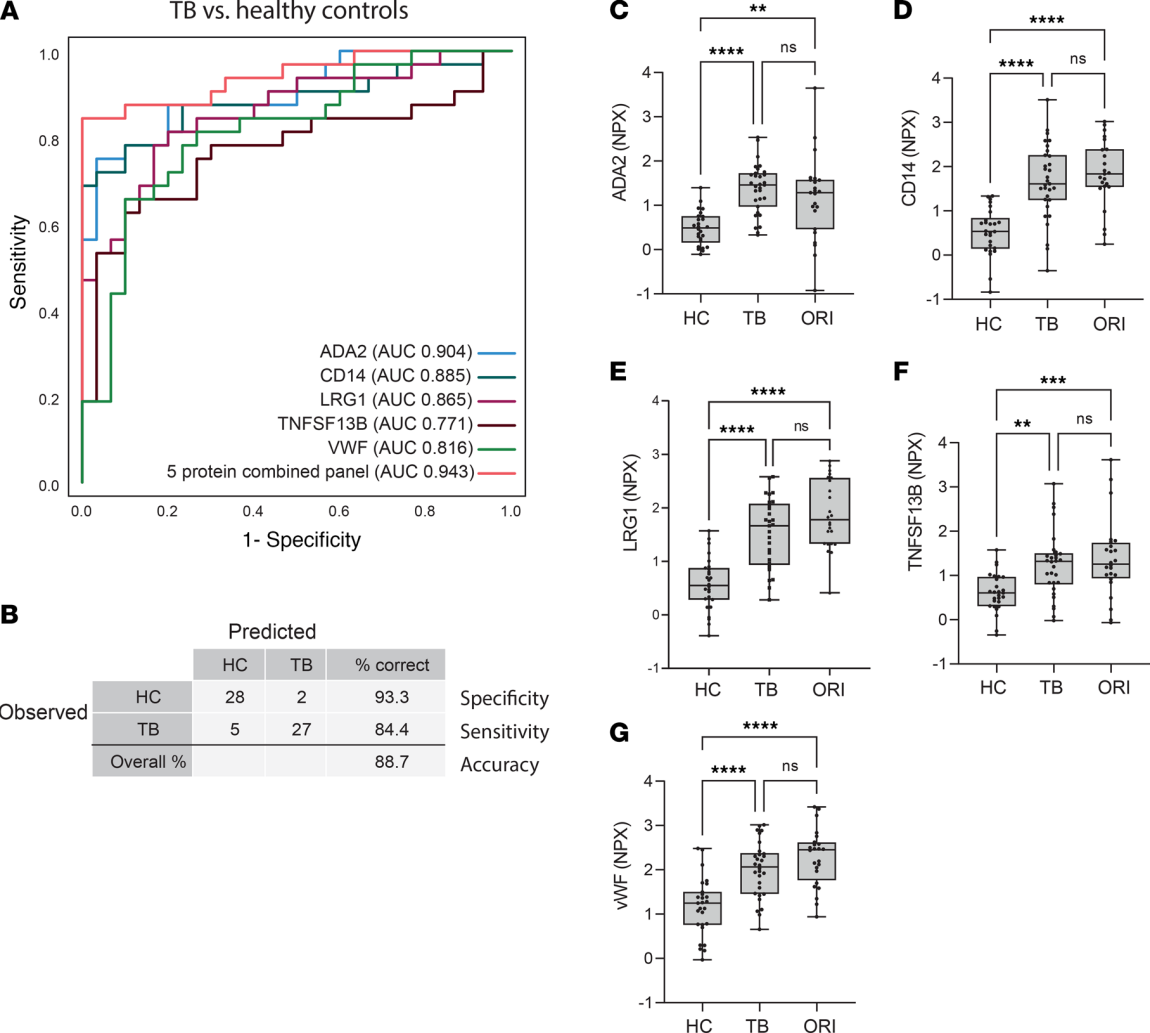
A 5-protein biomarker panel distinguishes pulmonary TB from healthy controls. (**A**) Receiver operating characteristic (ROC) curve of the best-performing 5-biomarker combination distinguishing pulmonary TB from HCs, demonstrating an AUC of 0.943 (95% CI: 0.889–1.000). (**B**) Classification grid illustrating diagnostic performance of the 5-protein biomarker panel in the validation cohort demonstrating a sensitivity of 84.4% (95% CI 67.3%–94.3%), specificity of 93.3% (95% CI: 75.8%–98.8%), and correct classification in 88.7% of cases. (**C**–**G**) Box-and-whisker plots of the 5 constituent proteins significantly differentially expressed in TB compared with HCs by proximity extension assay. Boxes show median values and interquartile ranges and whiskers show minimum to maximum values. Statistical differences were calculated using 1-way ANOVA with Tukey’s multiple-comparisons test for data with a Gaussian distribution and Kruskal-Willis test with Dunn’s multiple-comparison test for nonparametrically distributed data. NPX, normalized protein expression (log_2_ scale); AUC, area under the curve; HC, healthy control (*n* = 30); TB, tuberculosis (*n* = 32); ORI, other respiratory infection (*n* = 26); ADA2, adenosine deaminase 2; CD14, monocyte differentiation antigen CD14; LRG1, leucine-rich α-2-glycoprotein; TNFSF13B, tumor necrosis factor ligand superfamily member 13B; vWF, von Willebrand factor. NS, *P* > 0.05; **P ≤ 0.01, ***P ≤ 0.001; ****P ≤ 0.0001.

Diagnostic performance of individual markers was evaluated using receiver operating characteristic (ROC) curves. ADA2 and CD14 were the best-performing individual markers, distinguishing TB from HCs with an area under the curve (AUC) of 0.904 and 0.885, respectively (Figure 9A). Biomarker combinations were then evaluated using CombiROC analysis, to identify panels with a minimum diagnostic sensitivity of 90% and specificity of 70%, thereby meeting WHO TPP characteristics of a triage test for TB. ROC curves were generated following binary logistic regression of biomarker combinations to classify TB from HC samples. A 5-protein panel comprising ADA2, CD14, LRG1, TNFSF13B, and vWF gave an AUC of 0.943 (95% CI: 0.889–1.000; Figure 9A). This panel accurately classified patients in 88.7% of cases, with a sensitivity of 84.4% (95% CI: 67.3%–94.3%) and specificity of 93.3% (95% CI: 75.8%–98.8%; Figure 9B) at a probability cutoff of 0.5 or greater. Analysis of each analyte individually showed that they were highly significant compared with HCs, but were also significantly increased in ORI cases, suggesting they are not TB specific and are best suited for a rule-out test (Figure 9, C–G).

### A 6-protein panel differentiates pulmonary TB from ORI.

**Figure 10:**
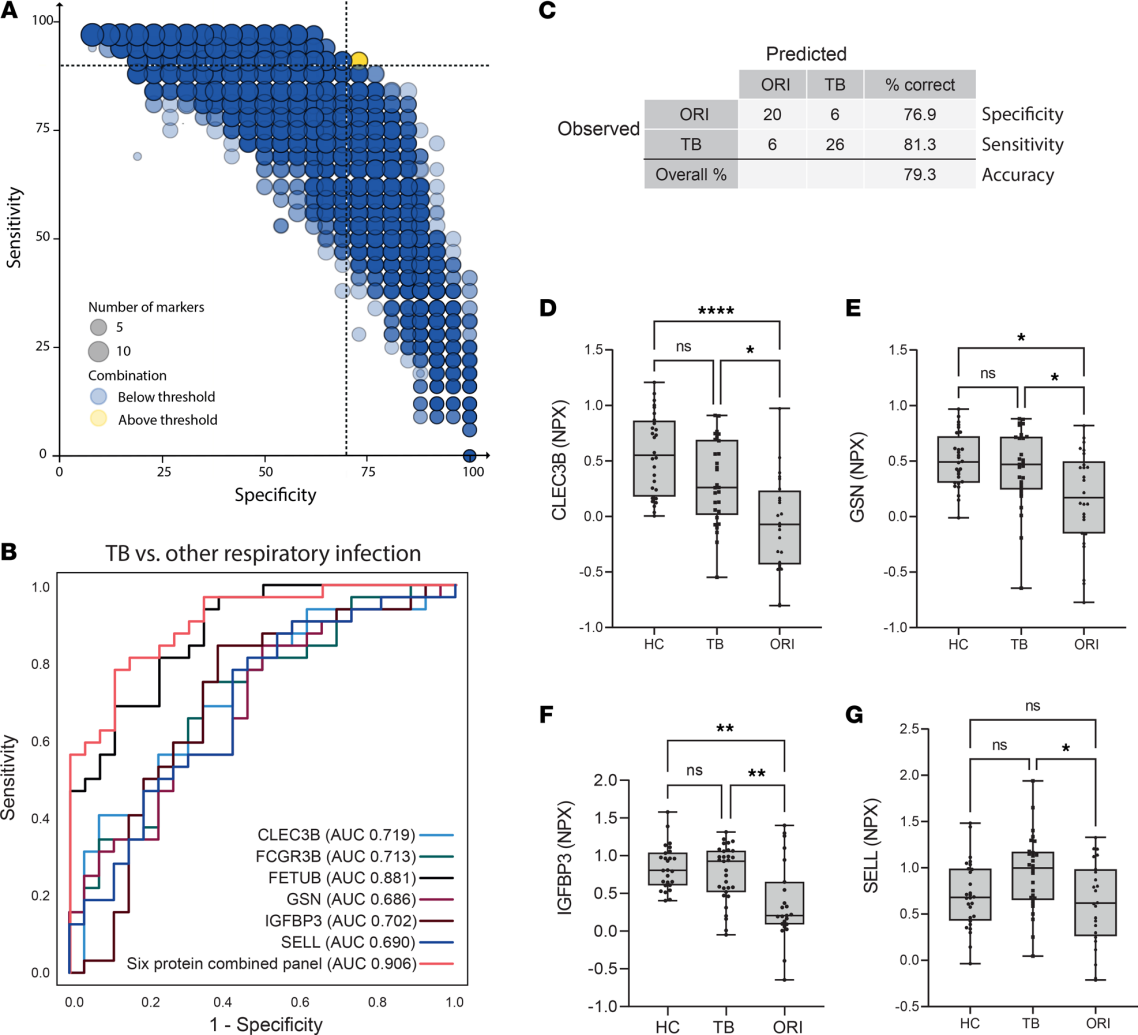
A 6-protein biomarker panel distinguishes pulmonary TB from other respiratory infections. (**A**) Bubble plot of possible protein combinations within the 14 proteins showing significant differential expression between TB and ORI groups, generated using the CombiROC R package. Dotted lines at 90% sensitivity and 70% specificity corresponding to the WHO Target Product Profile for a triage test for active TB. (**B**) Receiver operating characteristic (ROC) curve of best-performing biomarker combination and constituent proteins. The 6-protein combined panel AUC = 0.906 (95% CI: 0.83–0.908). (**C**) Classification grid illustrating diagnostic performance of the 6-protein biomarker panel in the validation cohort demonstrating a sensitivity of 81.3% (95% CI: 63.0%–92.1%), specificity of 76.9% (95% CI: 56.0%–90.2%), and correct classification in 79.3% of cases. (**D**–**G**) Box-and-whisker plots of protein biomarkers significantly differentially expressed in TB compared with other respiratory infections by proximity extension assay. Box-and-whisker plots of FCGR3B and FETUB are shown in Figure 8. Boxes show mean values and interquartile ranges and whiskers show minimum to maximum values. NPX, normalized protein expression (log_2_ scale); AUC, area under the curve; HC, healthy control; TB, tuberculosis; ORI, other respiratory infections; CLEC3B, tetranectin; GSN, gelsolin; IGFBP3, insulin-like binding protein 3; SELL, L-selectin; FCGR3B, low-affinity immunoglobulin receptor 3B; FETUB, fetuin-B. NS, *P* > 0.05; *P ≤ 0.05; **P ≤ 0.01, ****P ≤ 0.0001.

CombiROC analysis of the 14 significantly differentially expressed proteins between TB and ORI was performed to identify the best-performing panel (Figure 10A). The combination above the defined threshold comprised FCGR3B, FETUB, GSN, IGFBP3, SELL, and CLEC3B (Figure 10B). This combination had an AUC of 0.906 (95% CI: 0.8333–0.908), correctly classifying 79.3% of cases with a sensitivity of 81.3% (95% CI: 63.0%–92.1%) and a specificity of 76.9% (95% CI: 56.0%–90.2%; Figure 10C) at a probability cutoff of 0.5 or greater. Analysis of individual analytes demonstrated that they were significantly different between TB and ORI (Figure 10, D–G), but only FCGR3B and FETUB were also significantly different from HCs (Figure 8, B and C).

### Integration of top-performing analytes into a single panel provides differentiation of TB from both HCs and patients with ORI.

**Figure 11:**
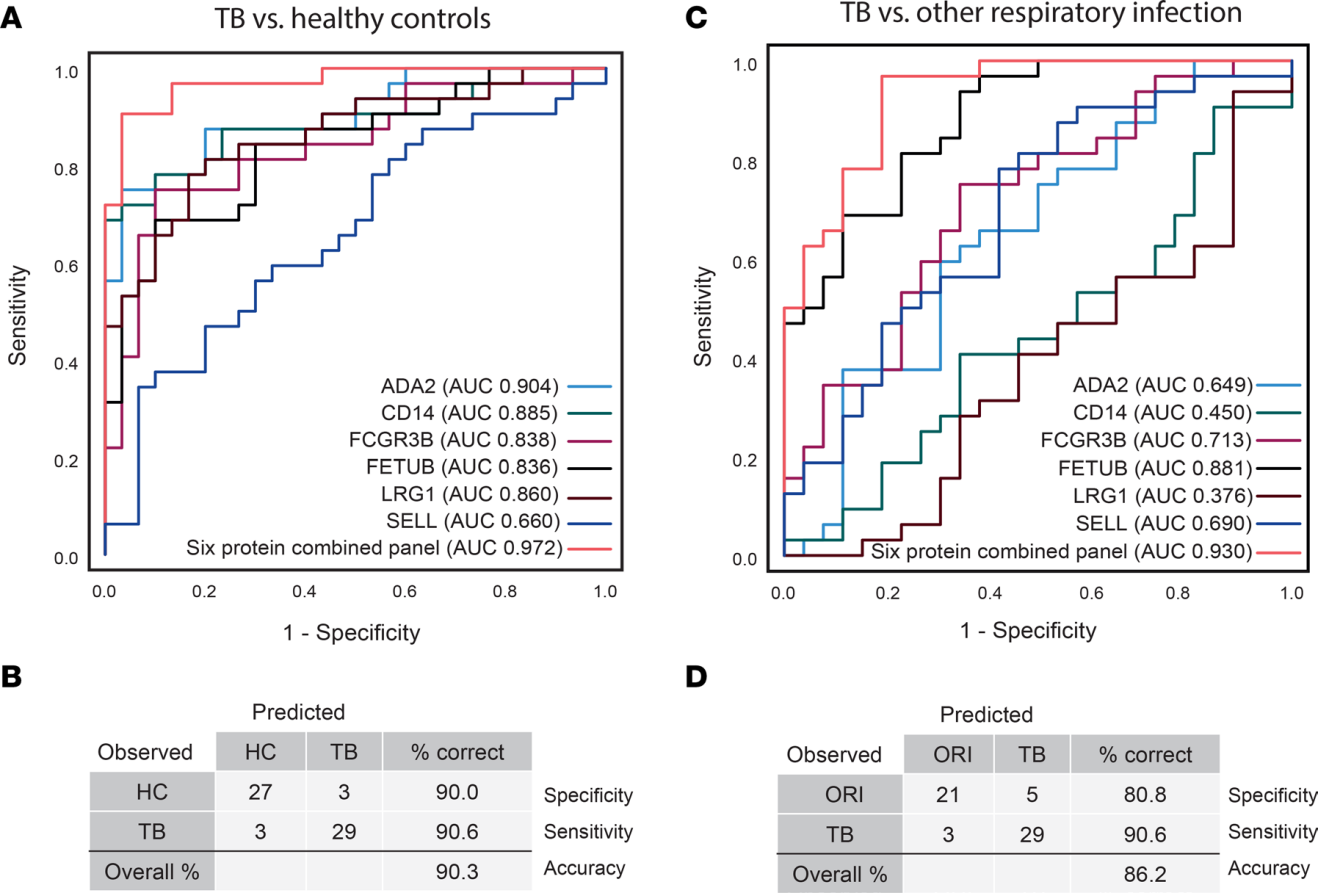
A final combined 6-protein panel discriminates patients with TB from both healthy controls and other respiratory infections. (**A**) ROC curve and (**B**) classification grid of the final 6-protein panel comprising FCGR3B, FETUB, LRG1, ADA2, CD14, and SELL, demonstrating discrimination of patients with TB from healthy controls (AUC 0.972 [95% CI: 0.937–1.000], sensitivity 90.6% [95% CI: 73.8%–97.5%], specificity 90.0% [95% CI: 72.3%–97.4%]). (**C**) ROC curve and (**D**) classification grid of the final 6-protein panel discriminating patients with TB from patients with other respiratory infections (AUC 0.930 [95% CI: 0.867–0.993], sensitivity 90.6% [95% CI: 66.5–96.7], specificity 80.8% [95% CI: 68.2–94.5]). All ROC curves and classification grids were generated using SPSS v28.0.1.0 after binary logistic regression for combined proteins. AUC was calculated under nonparametric assumption. TB was set as the positive test outcome and the test direction such that a larger test result indicates a more positive test. ROC, receiver operating characteristic; ADA2, adenosine deaminase 2; CD14, monocyte differentiation antigen; FCGR3B, low-affinity immunoglobulin receptor 3B; FETUB, fetuin-B; LRG1, leucine-rich α-2-glycoprotein; SELL, L-selectin; TB, tuberculosis; HC, healthy control; ORI, other respiratory infections.

A universal biomarker panel capable of differentiating individuals with TB from both healthy individuals and individuals with ORI would be more widely applicable to different population testing scenarios. Therefore, biomarker panel combinations were explored using proteins from each of the differentiating panels to identify the best-performing universal biomarker panel for both group comparisons. A 6-protein marker combination of FCGR3B, FETUB, LRG1, ADA2, CD14, and SELL performed very well for both group comparisons: TB versus HCs with an AUC of 0.972 (95% CI: 0.937–1.000), sensitivity of 90.6% (95% CI: 73.8%–97.5%), specificity of 90.0% (95% CI: 72.3%–97.4%; Figure 11, A and B); and TB versus ORI with an AUC of 0.930 (95% CI: 0.867–0.993), sensitivity of 90.6% (95% CI: 66.5%–96.7%), and specificity of 80.8% (95% CI: 68.2%–94.5%) (Figure 11, C and D) at a probability cutoff of 0.5 or greater. Performance of this final 6-protein panel was also evaluated by sex, as the discovery set had been exclusively male. This analysis confirmed the diagnostic performance of markers in male patients, and notably exceeded this in female patients (Supplemental Figure 9).

### The 6-protein panel discriminates TB from HCs and patients with ORI in a second independent patient cohort.

**Figure 12:**
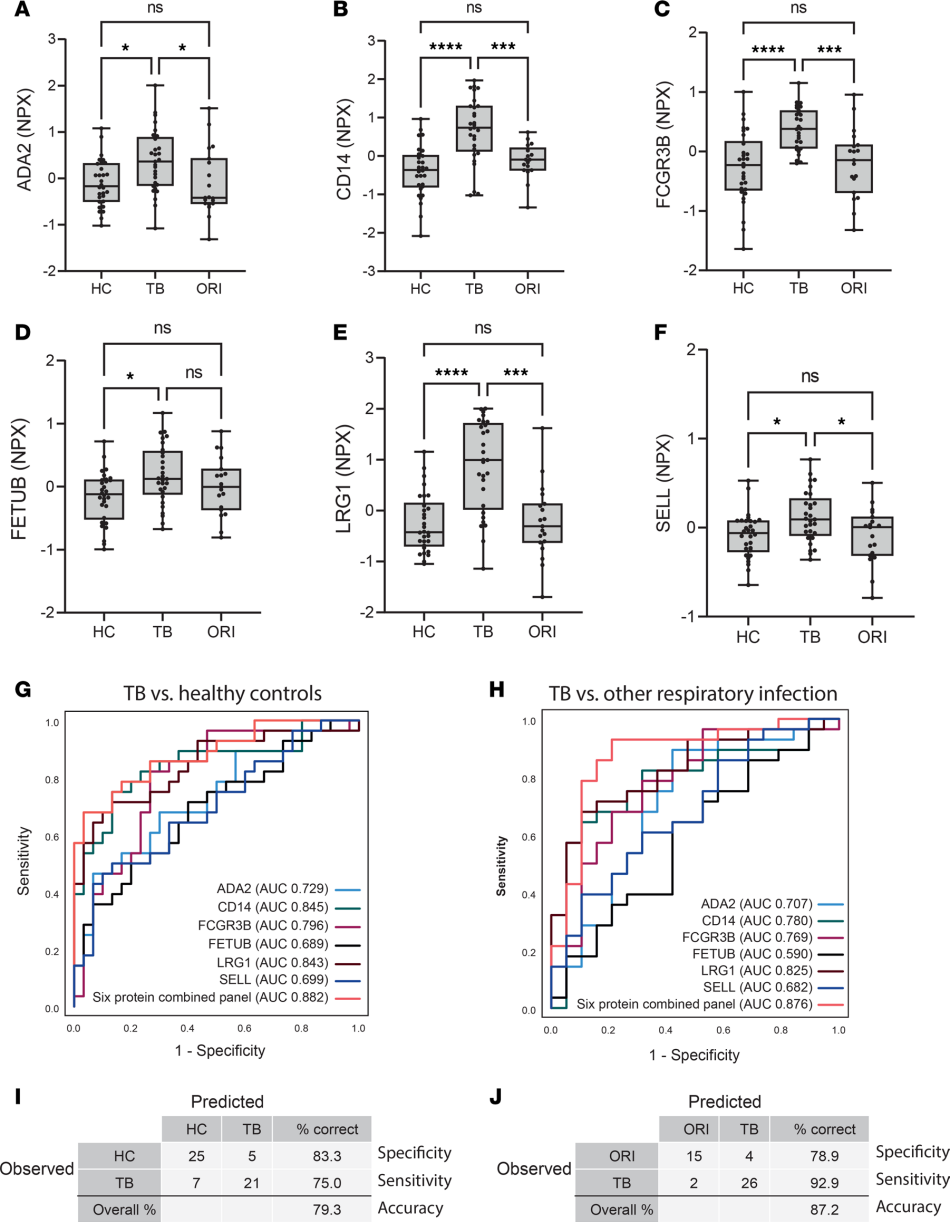
The final 6-protein panel differentiates TB from both HC and ORI in a separate clinical cohort. (**A**–**F**) Box-and-whisker plots of the 6 proteins in the panel in pulmonary TB compared with HC and ORI by proximity extension assay. Boxes show median values and interquartile ranges and whiskers show minimum to maximum values. Statistical differences were calculated using 1-way ANOVA with Tukey’s multiple-comparison test for data with a Gaussian distribution and Kruskal-Willis test with Dunn’s multiple-comparison test for nonparametrically distributed data. (**G**) Receiver operating characteristic (ROC) curve of the 6-protein panel distinguishing pulmonary TB from HCs. The 6-protein combined panel AUC = 0.882 (95% CI: 0.796–0.968). Full coordinates in Supplemental Table 16. (**H**) ROC curve of the 6-protein panel distinguishing pulmonary TB from ORI, AUC = 0.876 (95% CI: 0.765–0.987). Full coordinates in Supplemental Table 17. (**I**) Classification grid illustrating diagnostic performance of the 6-protein panel distinguishing pulmonary TB from HCs, demonstrating a sensitivity of 75.0% (95% CI: 54.8%–88.6%), specificity of 83.3% (95% CI: 64.5%–93.7%), and correct classification in 79.3% of cases in this cohort. (**J**) Classification grid illustrating diagnostic performance of the 6-protein panel distinguishing pulmonary TB from other respiratory infection, demonstrating a sensitivity of 92.9% (95% CI: 75.0%–98.8%), specificity of 78.9% (95% CI: 53.9%–93.0%), and correct classification in 87.2% of cases in this cohort. All ROC curves and classification grids were generated using SPSS v28.0.1.0 after binary logistic regression for combined proteins. AUC was calculated under nonparametric assumption. TB was set as the positive test outcome and the test direction such that a larger test result indicates a more positive test. NPX, normalized protein expression (log_2_ scale); AUC, area under the curve; HC, healthy control (*n* = 30); TB, tuberculosis (*n*= 29); ORI, other respiratory infection (*n* = 19); ADA2, adenosine deaminase 2; CD14, monocyte differentiation antigen CD14; LRG1, leucine-rich α-2-glycoprotein; TNFSF13B, tumor necrosis factor ligand superfamily member 13B; vWF, von Willebrand factor. NS, *P* > 0.05; *P ≤ 0.05; ***P ≤ 0.001; ****P ≤ 0.0001.

An antibody-based PEA was then used to test the diagnostic performance of the final 6-protein combination in a further independent cohort of plasma samples collected in South Africa (Supplemental Table 14) (28). Samples were selected from HIV-negative individuals with microbiologically confirmed pulmonary TB (TB, *n* = 29), HCs (*n* = 30) and individuals presenting with symptoms of pulmonary TB but were negative for *M*. *tuberculosis* on subsequent microbiological testing (ORI, *n* = 19), as outlined in Supplemental Table 13. Alternative diagnoses were not microbiologically confirmed in the ORI group due to the resource-limited healthcare setting, but symptoms were consistent with TB. Significantly elevated circulating levels of all 6 proteins in the panel were confirmed (Figure 12, A–F, and Supplemental Table 15). Analysis of the diagnostic performance of the 6-protein combined panel demonstrated diagnostic specificity for differentiation of TB from both HCs (AUC 0.883 [95% CI: 0.796 – 0.968], sensitivity of 75.0% [95% CI: 54.8%–88.6%], and specificity of 83.3% [95% CI: 64.5%–93.7%]; Figure 12, G and I); and ORI (AUC 0.876 [95% CI: 0.765–0.987], sensitivity of 92.9% [95% CI: 75.0%–98.8%], and specificity of 78.9% [95% CI: 53.9%–93.0%]; Figure 12, H and J) at a probability cutoff of 0.5 or greater. Diagnostic performance of the final 6-protein panel was also tested in both patient cohorts against a combined group of both HCs and ORI, which confirmed preserved specificity of performance (Supplemental Figure 10).

## Discussion

TB remains a global catastrophe, and a fundamental issue in controlling the pandemic is the limitations of the diagnostic process, resulting in an estimated 4.2 million missed cases in 2022 (3). This diagnostic gap leads to ongoing transmission, morbidity and mortality, and long-term strain on healthcare systems (6, 29). A novel diagnostic assay with high levels of accuracy would be transformative, permitting population screening to find the missing millions and thereby break the cycle of transmission (30). Indeed, mass screening is being increasingly advocated as a central pillar to control the TB pandemic (3, 7, 31–34). However, this requires new tools that are fit for purpose, utilizing non–sputum-based approaches, but the incomplete understanding of potential plasma biomarkers has considerably limited progress (3).

Here, we utilized a non-depletion quantitative proteomics approach to generate what we believe is the most detailed description of the plasma proteome of TB to date. Complementary bioinformatic analysis using linear modeling and correlation network analysis identified 118 differentially expressed proteins compared with HCs. A large subset of biomarkers were successfully validated in a separate clinical cohort by an antibody capture approach, demonstrating that analytes can progress to different platforms and overcome this hurdle that may limit translation of proteomics-discovered biomarkers. Four TB-specific biomarkers, FETUB, FCGR3B, GGH, and SERPIND1, were increased in TB patients compared with both HCs and sick controls with ORI. Combinatorial analysis using a CombiROC approach identified a 6-protein biomarker panel that could distinguish active pulmonary TB from HCs and patients with ORI, achieving the TPP of the WHO (6). Further validation in a second independent cohort demonstrated statistically significant elevation of all 6 proteins in the plasma of TB patients and confirmation of high diagnostic performance of the combination panel, distinguishing active pulmonary TB from HCs and ORI. Our discovery proteomic protocol did not involve depletion steps, in contrast with many previous MS-based plasma proteomic studies in TB (10–12, 35, 36). Plasma depletion can remove proteins of potential biological interest that are associated with the target protein by noncovalent interactions (18–20). We employed complementary bioinformatic methodologies to identify candidate biomarkers, with *limma* employing Bayesian statistics (23), while WGCNA circumvented limitations of multiple comparisons by using unsupervised analysis methods to generate modules of coexpressed proteins that correlate with clinical traits (24). The 118 proteins identified by all 3 complementary approaches were considered the strongest biomarker candidates.

We identified numerous previously described biomarkers of TB such as CRP, LBP, serum amyloid A1 (SAA1), α-1-acid glycoprotein 1 (ORM1), and retinol-binding protein 4 (RBP4) alongside S100A8 and S100A9, the protein components of calprotectin. In addition, we identified several biomarkers that we believe have not previously been described, such as lymphocyte cytosolic protein 1 (LCP1), γ-glutamyl hydrolase (GGH), marginal zone B and B1 cell–specific protein (MZB1), and FETUB, including proteins not known to be secreted into the extracellular compartment, such as transcription termination factor 1 (TTF1). LCP1 is a leukocyte-specific actin-binding protein that is required for podosome formation and function in macrophages (37). LCP1 has been identified in the phagosomes of BCG-infected macrophages (38). GGH is a protease typically located in lysosomes, and serum GGH has been proposed to be a marker of oxidative stress (39). MZB1 aids peripheral B cell function and promotes secretions of IgM antibodies (40, 41). TTF1 is a multifunctional protein that usually localizes to the nucleolus (42) and regulates transcription of surfactant protein B (SFTPB) in type 2 alveolar cells (43, 44). SFTPB is also upregulated in our data set.

Lung matrix destruction and cavitation is a hallmark of pulmonary TB, which leads to morbidity, mortality, and increased disease transmission (45, 46). Our findings further highlight matrix turnover as a central process in TB. Gene ontology analysis of differentially expressed proteins showed that the extracellular matrix was the most significantly enriched cellular compartment; the most significantly enriched molecular functions were endopeptidase and peptidase inhibitor and regulator activity; and the highest proportion of significantly enriched biological processes related to proteolysis. The SERPINs are a large family of serine protease inhibitors (47) and 8 SERPINs were differentially regulated, with elevated SERPIND1 levels shown to have the highest specificity for TB. FETUB, a cysteine protease inhibitor, emerged as a key biomarker for pulmonary TB, but little is known about its pathological role. FETUB is part of a 9-protein prognostic risk score in lung adenocarcinoma (48) and plasma levels correlate with worsening lung function in chronic obstructive pulmonary disease (COPD) (49), suggesting plasma FETUB levels may relate to destructive lung pathology.

Pulmonary TB is characterized by excessive inflammation (50), and we identified numerous inflammation-related proteins such as CRP, S100A8, and S100A9. ADA2, CD14, and LRG1, part of the final 6-marker panel, have all been implicated in inflammatory responses. ADA2 induces the differentiation of monocytes to macrophages and stimulates macrophage and helper T cell proliferation (51); CD14 serves as a receptor for *M*. *tuberculosis* cell wall lipoarabinomannan (52, 53); while LRG1 is a marker for neutrophilic granulocyte differentiation, which we have previously shown to be elevated in the serum of patients with pulmonary TB (21). FCGR3A and FCGR3B, low-affinity immunoglobulin receptors, were also upregulated. These only differ by 1 amino acid, with FCGR3A expressed on NK cells and FCGR3B in monocytes and macrophages (54). FCGR3B upregulation was relatively specific for TB, not being upregulated in ORI. Complement components were also upregulated, including C2, C4B, C8B, CFB, C9, and CFHR5, demonstrating broad modulation of this inflammatory pathway in TB disease (55).

Among the significantly downregulated proteins, lipid metabolism featured strongly, enriched for the lipoprotein cellular compartment, lipid binding, and lipid inflammatory mediator–binding molecular functions. Lipid metabolism and systemic inflammation are inextricably intertwined (56), with eicosanoid-mediated inflammatory imbalance implicated in human TB (57). Leukotriene A4 hydrolase (LTA4H) is elevated in TB and has been implicated in the spatial organization of lipid signaling within TB lung granulomas by a proteomics approach (58), and regulates susceptibility to infection (59). Additionally, previous hypothesis-directed approaches have shown lower levels of cholesterol, HDL-C, and LDL-C levels in pulmonary TB patients compared with controls (60).

Differences in TB pathogenesis between ethnic groups has been recognized for over a century (61, 62), and ethnicity has been shown to be a powerful determinant of clinical TB phenotype, independent of *M*. *tuberculosis* strain lineage (63). We analyzed plasma samples from 2 geographical origins, South Africa and Peru, and identified differences in the plasma proteome by region both in HCs and in TB patients. Such geographical differences need consideration in developing new diagnostic tests (64). Reassuringly, our top candidate biomarkers were validated in an independent cohort of mixed ethnicity and sex, and the 6-protein biomarker panel in a further independent clinical cohort of mixed sex.

Previous studies have explored circulating biomarkers of TB disease utilizing diverse approaches. Luminex-based analysis of HIV-negative individuals from sub-Saharan African countries for prespecified analytes has identified a 2-protein panel and a 9-protein panel, both including CRP, that distinguish TB from other respiratory diseases, with comparatively high sensitivity, but lower specificity (14, 15). A Simoa ultrasensitive immunoassay comprising 4 host proteins and an antibody against TB antigen Ag85B was also able to discriminate between patients with TB and those with other respiratory diseases, but had lower performance characteristics than our biomarker panel, and, importantly, requires a specific reader (65). In another study, analysis by aptamer-based SOMAscan assays identified a 6-protein panel comprising cytoplasmic tryptophan-tRNA ligase (SYWC), kallistatin, C9, gelsolin, testican-2, and aldolase C (16), which could distinguish TB from non-TB samples with a similar sensitivity and specificity to our panel, though limited data were available regarding the patients that made up the non-TB group. Our unbiased discovery approach using geographically diverse populations demonstrates a robust method for the identification of protein biomarkers with higher specificity for differentiating TB disease in carefully phenotyped comparator groups of HCs and ORI. Evidently, the performance of our proposed biomarkers will require validation in additional cohorts, including patients with extrapulmonary TB and individuals with HIV coinfection, which present additional diagnostic challenges (66). An assay will be needed that meets the WHO ASSURED criteria for a point-of-care test for use in resource-limited settings, being affordable, sensitive, specific, user-friendly, rapid, equipment-free, and deliverable to those in need (67). Recent developments in integrated microfluidic systems may allow the translation of diagnostic panels into an immunoassay-based lab-on-a-chip system, that would have potential for near-patient use (6).

In summary, our integrated proteomics approach has identified TB-specific circulating biomarkers of disease among a group of 118 divergently regulated proteins identified through a rigorous bioinformatic pipeline. A 6-protein biomarker panel can discriminate individuals with active pulmonary TB from healthy individuals and from those with other bacterial or viral pulmonary infections, with potential for onward development into a point-of-care test suitable for mass population screening. The diagnostic potential of these protein biomarkers and panels require further validation in key clinical groups, such as HIV-coinfected individuals and in cohorts with high coprevalence of common comorbidities such as diabetes and COPD. Additionally, although our study focused on separating infection from TB, in future comparison with sarcoidosis, autoimmune pneumonias, or chronic fungal pneumonias in specific settings where these are prevalent will also be warranted. While future validation in different cohorts and development of a near-patient assay represent significant future hurdles, we propose that these findings provide critical knowledge to develop an initial screening assay that can be used to triage patients to pathways involving more expensive confirmatory testing for TB (7, 68). Such active case finding will help to close the case-detection gap that is fueling the ongoing TB pandemic.

## Methods

### Sex as a biological variable

Sex has been carefully considered as a biological variable in this investigation. For the discovery plasma MS, only samples from male patients were used, as males exhibit the most florid pulmonary TB pathology. For both validation cohorts, samples from males and females were tested, and ratios are presented in Supplemental Tables 11 and 14.

### Study participants

Participants in the discovery experiment were recruited in 2 separate cohorts. The South African cohort was recruited at Ubuntu TB/HIV clinic in Cape Town from June 2012 to February 2014 and were of Black African ethnicity (28). Written informed consent was provided. The diagnosis of active TB was based on sputum smear or culture positivity, GeneXpert results where available, and chest radiograph findings. For HCs, sputum samples were smear and culture negative for acid-fast bacilli. The Peruvian cohort was recruited at clinics in Lima, Peru during 2015. The diagnosis of TB was based on TB symptoms, sputum smear and culture positivity, and chest radiograph findings. HCs were QuantiFERON negative, excluding coincidental latent TB infection (LTBI). Plasma samples from male HIV-negative participants were randomly selected for the discovery experiment from either cohort if they were between the ages of 18 and 50 years old and had a BMI between 16 and 26 and there was sufficient sample for analysis. Exclusion criteria included anemia (Hb ≤ 8 g/dL), significant renal impairment (creatinine ≥ 150 μm/L), significant hepatic disease (ALT ≥ 80 IU/L), and known malignancy or diabetes mellitus. Patients with active TB had not yet commenced treatment at the time of plasma sampling.

Participants in validation cohort 1 were from the UK-based MIMIC cohort of mixed ethnicity. Patients were recruited between June 2014 and February 2017. All participants in the MIMIC study were UK residents at the time of sample collection and were HIV-negative. HCs were asymptomatic, without a history of previous TB disease, TB contact or travel to a high TB prevalence area, and no evidence of LTBI in IFN-γ release assay testing. Active pulmonary TB cases were symptomatic individuals with microbiologically confirmed TB by either sputum smear, sputum culture, or positive PCR for *M*. *tuberculosis*. Individuals with ORI were symptomatic with microbiologically confirmed respiratory tract infection caused by a pathogen other than *M*. *tuberculosis*, without a history of previous active TB. The causative agents in this group comprised influenza virus A and B, respiratory syncytial virus, human metapneumovirus, *Streptococcus pneumoniae*, *Staphylococcus aureus*, and *Mycoplasma pneumoniae*. All participants in validation cohort 2 were resident in Khayelitsha, Cape Town at the time of sample collection, were of Black African ethnicity, and HIV-uninfected. The diagnosis of TB was based on TB symptoms, sputum smear and culture positivity, and chest radiograph findings.

### Sample processing

For the discovery experiment, venous blood was collected in sodium heparin vacutainer tubes and plasma prepared according to standard operating procedures at the site of recruitment and stored at –80°C. Aliquots of 120 μL of plasma were liquid fixed with 380 μL of 7 M guanidine hydrochloride and 10% methanol and stored at –20°C until size exclusion chromatography. Aliquots of 20 μL of each plasma sample in the discovery experiment was combined to generate a master-pool sample to help mitigate batch effects across different proteomic experiments.

For the validation experiment in the MIMIC cohort, venous blood was collected in serum vacutainer tubes and serum prepared according to standard operating procedures at the site of recruitment and stored in 100 μL aliquots at –80°C. For PEA, serum samples were thawed, centrifuged for 10 minutes at 455*g*, and 40 μL per sample aliquoted into a 96-well plate and re-frozen at –80°C until analysis at the Oxford Genomics Centre.

### Discovery proteomic analysis

#### High-performance size-exclusion chromatography and protein digestion.

The methodology for high-performance size-exclusion chromatography has been previously described (21). Total protein lyophilized extracts from each plasma segment were reconstituted with 0.5 M triethylammonium bicarbonate and 0.05% sodium dodecyl sulphate and sonicated on ice. Following centrifugation at 16,000*g* for 10 minutes at 4°C, protein content was estimated using a Nanodrop ND-1000 spectrophotometer (Thermo Fisher Scientific) at 280 nm. Volume-adjusted 120 μg of protein was reduced with 2 μL of 50 mM Tris-2-carboxymethyl phosphine and incubated at 60°C for 1 hour. Samples were then alkylated using 1 μL of 200 mM methylmethane thiosulphonate and incubated for 10 minutes at room temperature. Protein digestion was conducted at a ratio of 1:40 enzyme/substrate with trypsin MS grade (Pierce, Thermo Fisher Scientific) overnight at 37°C in the dark.

#### iTRAQ labeling.

Isopropanol was added to iTRAQ labels to ensure more than 60% organic phase during sample labeling and each tag was added to the appropriate trypsinized sample. The master pool was labeled using tag 113, and the samples were block randomized to the remaining tags according to Supplemental Table 2. The labeling reaction was conducted for 2 hours at room temperature and the reaction stopped with 8 μL of 5% ammonium hydroxylamine. Samples were lyophilized and stored at –20°C until chromatographic separation.

#### Peptide fractionation.

Offline peptide fractionation was performed at high pH (0.08% NH_4_OH) using a C_4_ column (Kromasil, 3.5 μm, 2.1 mm × 150 mm) on a Shimazdu HPLC system. iTRAQ-labeled peptides were reconstituted and pooled with 100 μL of mobile phase and centrifuged at 16,000*g* at room temperature for 10 minutes. Supernatant was injected and separated at a flow rate of 0.3 mL/min at 30°C. Fractions were collected by peak detected at 215 nm. Peptide fractions were dried using a Speedvac concentrator (Thermo Fisher Scientific) and stored at –20°C until LC-MS/MS analysis. Highly hydrophilic and hydrophobic fractions from the extreme regions of the chromatographic traces were pooled and further cleaned using Gracepure SPE C18-AQ 100 mg/1 mL cartridges (Thermo Fisher Scientific).

#### MS analysis.

Peptide fractions were analyzed using a Dionex Ultimate UHPLC system coupled to a nano-ESI-LTQ-Velos Pro Orbitrap Elite mass spectrometer (Thermo Fisher Scientific). Online chromatographic separation of each peptide fraction was conducted using an AcclaimPepMap RSLC C18 nanoViper column (Thermo Fisher Scientific; 2 μm, 75 μm × 25 cm). This was retrofitted to a PicoTip emitter (New Objective, FS360-20-10-D-20-C7) for injection into the mass spectrometer. MS characterization of eluting peptides was conducted between 380 and 1500 *m*/*z*. The top 10 +2 and +3 precursor ions were further characterized by tandem MS (MS/MS). Higher energy collisional dissociation (HCD) and collision-induced dissociation (CID) fragmentation for each of the collected fractions was performed.

Full MS scans and MS/MS scans were acquired at a resolution of 30,000 full width at half maximum (FWHM) for set C segments 1–3, and 60,000 FWHM for all further plasma segments. Data were acquired using Xcalibur software (Thermo Fisher Scientific). Conditions for ionization, CID and HCD fragmentation, and ion detection for this method have been previously reported (69).

#### MS data processing.

Target decoy searching of raw mass spectra was conducted with Proteome Discoverer v2.4 (Thermo Fisher Scientific). Sequest HT was used for target decoy search for tryptic peptides, allowing 2 missed cleavages, 10 ppm mass tolerance, and a minimum peptide length of 6 amino acids. Dynamic modifications of oxidation (M), deamidation (N, Q), and phosphorylation (S, T, Y) and static modifications of iTRAQ 8plex (N-terminus, K) and meythylthio (C) were permitted. Fragment ion mass tolerance was 0.02 Da for HCD-generated spectra and 0.5 Da for CID-generated spectra. Percolator was set to a concatenated strategy for target decoy selection with a strict FDR target of 0.01 and relaxed FDR target of 0.05. Spectra were searched against a concatenated FASTA file comprising the UniProtKB SwissProt human proteome and the reference *M*. *tuberculosis* H37Rv proteome (SwissProt and TrEMBL). Unique peptide spectrum matches were taken through to consensus workflow allowing a 50% co-isolation threshold and a signal-to-noise ratio of 3. Normalization was to total peptide amount and scaling was to controls average. This scaling enabled a multi-consensus workflow to generate grouped protein abundances across all 4 plasma segments for each experimental set. Protein abundances were imported to R (http://www.rstudio.com/) for log_2_ transformation, median normalization, data visualization, and bioinformatic analysis. Data from plasma samples from TB patients labeled with iTRAQ tags 118 and 121 in experimental set C were excluded from further analysis at this stage due to failure of normalization (tag 118) and clustering with the control group (121). Clinically, the latter patient had microbiologically confirmed pulmonary TB, but minimal chest x-ray changes and a normal CRP.

### Validation proteomic analysis

Serum samples from the MIMIC cohort were thawed and centrifuged at 15,000*g* for 10 minutes at 4°C. Serum was aliquoted onto 96-well PCR plates and transported on dry ice to the Oxford Genomics Centre for analysis. PEA was performed as per the proteomic method that has been previously described (70) using Olink Explore Cardiometabolic and Inflammation II panels. Each assay has been extensively validated for limit of detection, measurement ranges, precision, reproducibility, and specificity as detailed at https://olink.com/our-platform/assay-validation/#explore

### Statistics

#### Discovery proteomics.

Differentially expressed proteins were identified using linear modeling with the R package *limma* (23), including FDR correction for multiple comparisons and network correlation analysis using the R package WGCNA (24). The *limma* package was applied to combined data from each plasma segment and on multi-consensus analyses, following adjustment for experimental batch effects using the R package ComBat (22). WGCNA was applied to ComBat-adjusted data for combined multi-consensus analyses. WGCNA was used to determine clusters of highly correlated proteins (color modules) and explore their correlation with phenotypic traits. Module significance is expressed as a correlation score with statistical significance. Gene ontology enrichment analysis was conducted using ShinyGO (71), with all proteins identified from the discovery experiment as a background proteome. Only gene ontology terms with an FDR-adjusted *P* value of less than 0.05 were considered. Graphical visualizations of the enrichment analysis were generated using the R package clusterProfiler (72) for cnet plots and GOplots for chord plots.

#### Validation proteomics.

Differences in protein expression between groups for PEA measurements were analyzed using GraphPad Prism v9. Data distributions were examined for normality and differences analyzed by 1-way ANOVA if Gaussian distribution was found. For nonparametrically distributed data, differences between groups were analyzed using the Kruskal-Wallis method with Dunn’s test for multiple comparisons. A *P* value of less than 0.05 was considered statistically significant. Combinatorial performance of biomarkers was assessed using the R package CombiROC (73). ROC curves for clinical group classification were then explored for the best performing biomarker panels following binary logistic regression using SPSS v28.0.1.0 (IBM Statistics).

### Study approval

All clinical studies were conducted according to the Declaration of Helsinki principles. All participants gave written informed consent. The South African cohort was recruited under University of Cape Town Research Ethics Committee approval (HREC, REF 516/2011). Enrollment of participants in the Peruvian study was approved by the Universidad Peruana Cayetano Heredia Institutional Review Board (SIDISI 65314). University of Southampton Ethics and Research Governance approval was given for transporting samples to the UK for analysis (approval 17758). The MIMIC study was approved by the National Research Ethics Service Committee South Central (REF 13 SC 0043).

### Data availability

The mass spectrometry proteomics data have been deposited to the ProteomeXchange Consortium via the PRIDE partner repository with the data set identifier PXD051070 (74). Selected PEA data are available in Supplemental Tables 13 and 15. Values for all data points shown in graphs are reported in the Supporting Data Values file. Further data and analysis code are available from the corresponding author on request.

## Author contributions

HS performed 6 of the 12 discovery proteomic experiments, analyzed and integrated the data from all 12 discovery data sets, performed and optimized the subsequent bioinformatic analysis, managed the MIMIC sample cohort, directed the validation proteomics analysis for both cohorts, performed statistical analysis of the validation data sets, and drafted the manuscript. DGB and PE designed the discovery experiment. DGB performed high-performance size-exclusion chromatography of all discovery plasma samples, 6 of the 12 discovery proteomic experiments, optimized the wet lab proteomic method, and provided R scripts for protein abundance normalization, *limma*, ComBat, and PCA. NFW and RJW recruited the South African cohorts and provided clinical annotation. CUG recruited the Peruvian cohort and provided clinical annotation. MT designed the MIMIC study, recruited the MIMIC cohort (Southampton site), and provided clinical annotation. DGB, AM, and SDG provided expertise in the plasma proteomic protocol. AFV, CHW, and MN provided expert insight into bioinformatic analysis and AFV provided the R scripts for WGCNA. LBT and SM provided expert insight into wet lab methodology and useful discussions throughout the project. PHW, GR, and PP performed the PEA analysis. HS and PE secured funding for the project. PE was involved in the study design, provided oversight to the project, and contributed to the manuscript writing and editing. All authors reviewed the manuscript, provided intellectual input, and approved the final version.

## Supplementary Material

Supplemental data

ICMJE disclosure forms

Supplemental tables 1-17

Supporting data values

## Version 1 03/21/2024

In-Press Preview

## Version 2 04/22/2024

Electronic publication
