# Supplementary material for: Integrated plasma proteomics identifies tuberculosis-specific diagnostic biomarkers
Source: JCI Insight. 2024 Mar 21;9(8):e173273. doi: 10.1172/jci.insight.173273 (PMC11141874; doi:10.1172/jci.insight.173273)
Supplement: Supplemental data [file jciinsight-9-173273-s226.pdf]

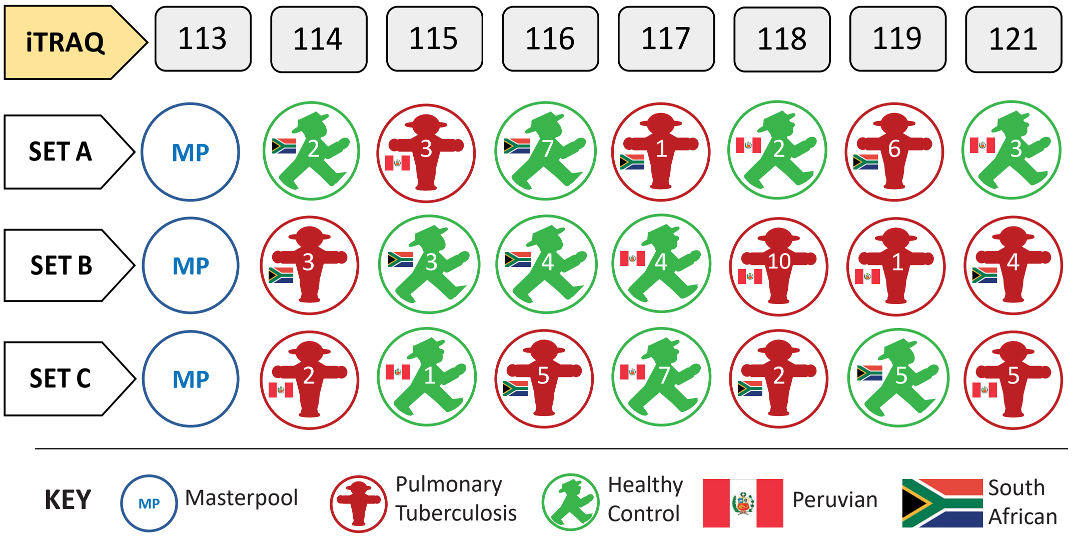

S1: Block randomised design of discovery proteomics experiment.
The design comprised three experimental sets: A, B & C. Peptides from each sample were iTRAQ-labelled following trypsin digestion according to this block randomised design. Each experimental set contained a bridging masterpool plasma sample which was labelled with iTRAQ tag 113 and either 3 or 4 plasma samples from healthy controls and individuals with pulmonary TB.

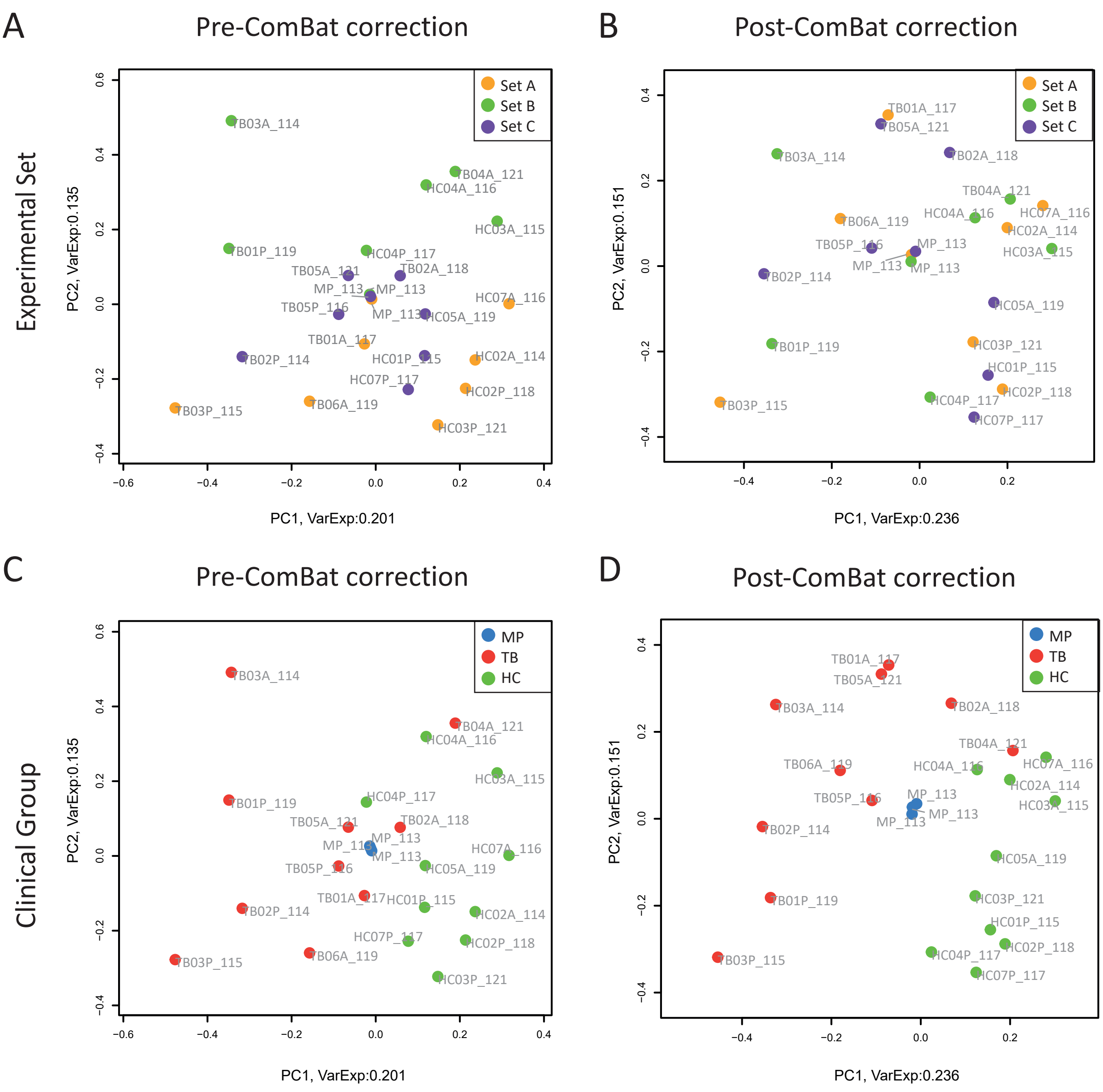

S2: Adjustment for batch effects between experimental sets.

Principal component analysis (PCA) depicting batch effect correction using the R package ComBat. PCA of protein abundances by experimental set before (A) and after (B) ComBat correction. PCA of protein abundances by clinical group before (C) and after (D) ComBat correction.

PC1 principal component one; PC2 principal component 2; VarExp explained variance; MP masterpool; TB pulmonary tuberculosis; HC healthy control.

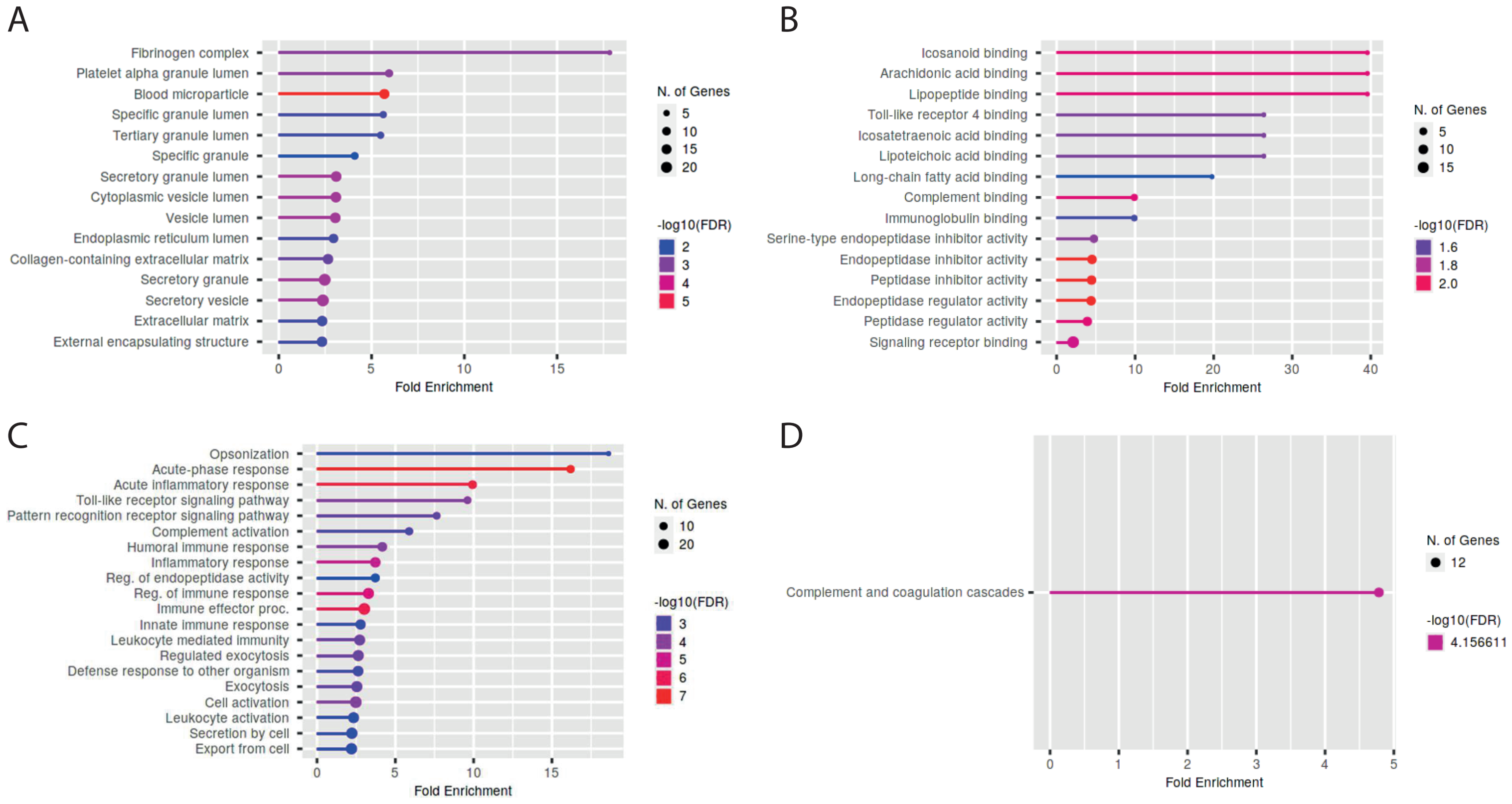

S3: Gene ontology analysis of significantly upregulated proteins.
Lollipop plots displaying fold enrichment and significance as false discovery rate (FDR) of ontology terms for (A) cellular compartment (B) molecular function (C) biological process and (D) KEGG pathways of upregulated proteins. The length of the lollipop is the fold enrichment of the pathway, the size of lollipop head indicates the number of proteins in the input dataset that are found within the pathway and the colour indicates the statistical significance of the enrichment. Gene ontology enrichment performed using ShinyGO against the background of the entire plasma proteome identified from discovery mass spectrometry proteomics.

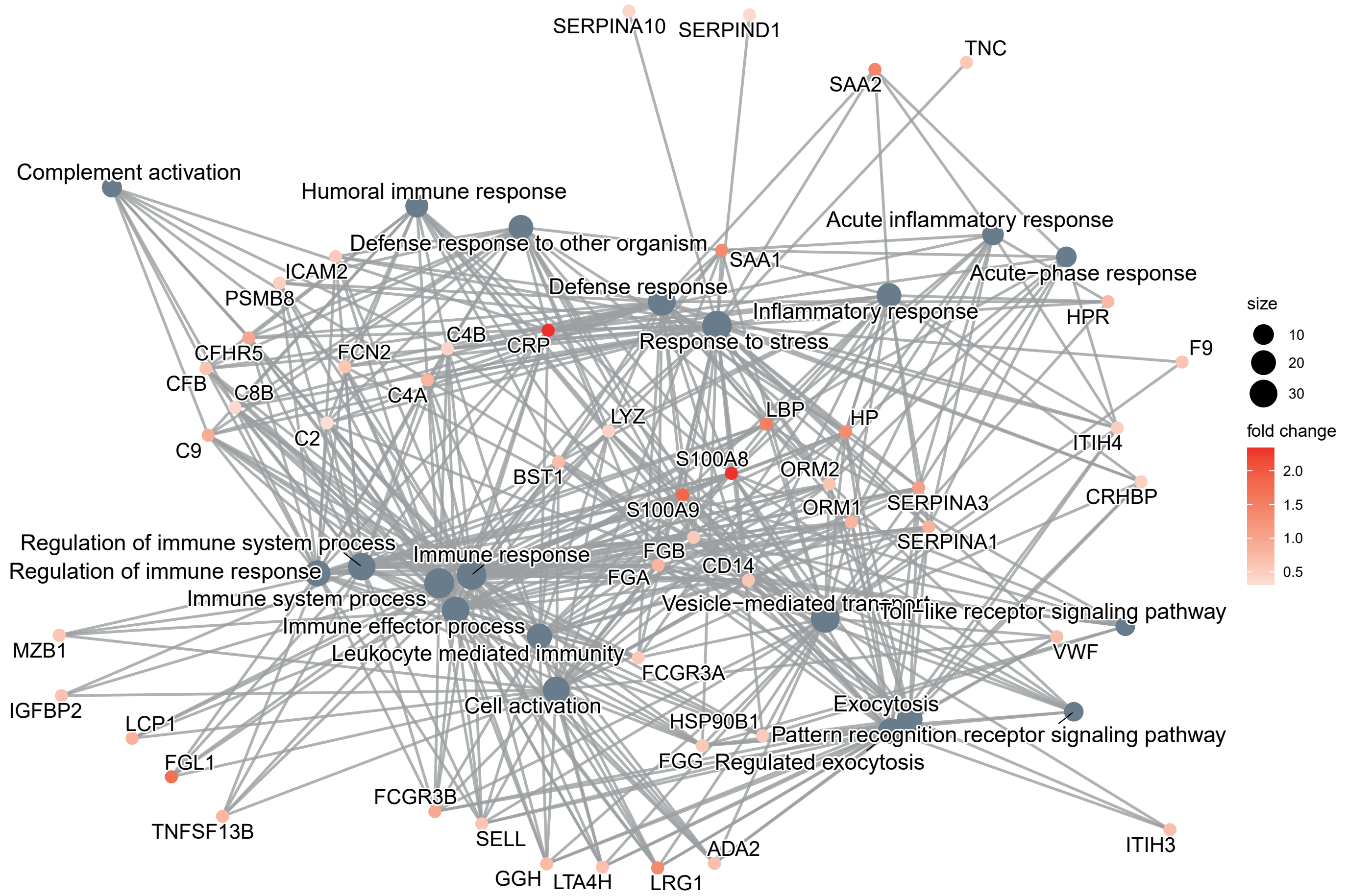

S4: Concept network plot of significantly upregulated proteins and their enriched biological processes.
Plot generated from ShinyGO enrichment by biological process of upregulated proteins using the cnetplot function in the R package GOplots. The top 20 most enriched pathways are displayed linked to their relevant differentially expressed proteins.

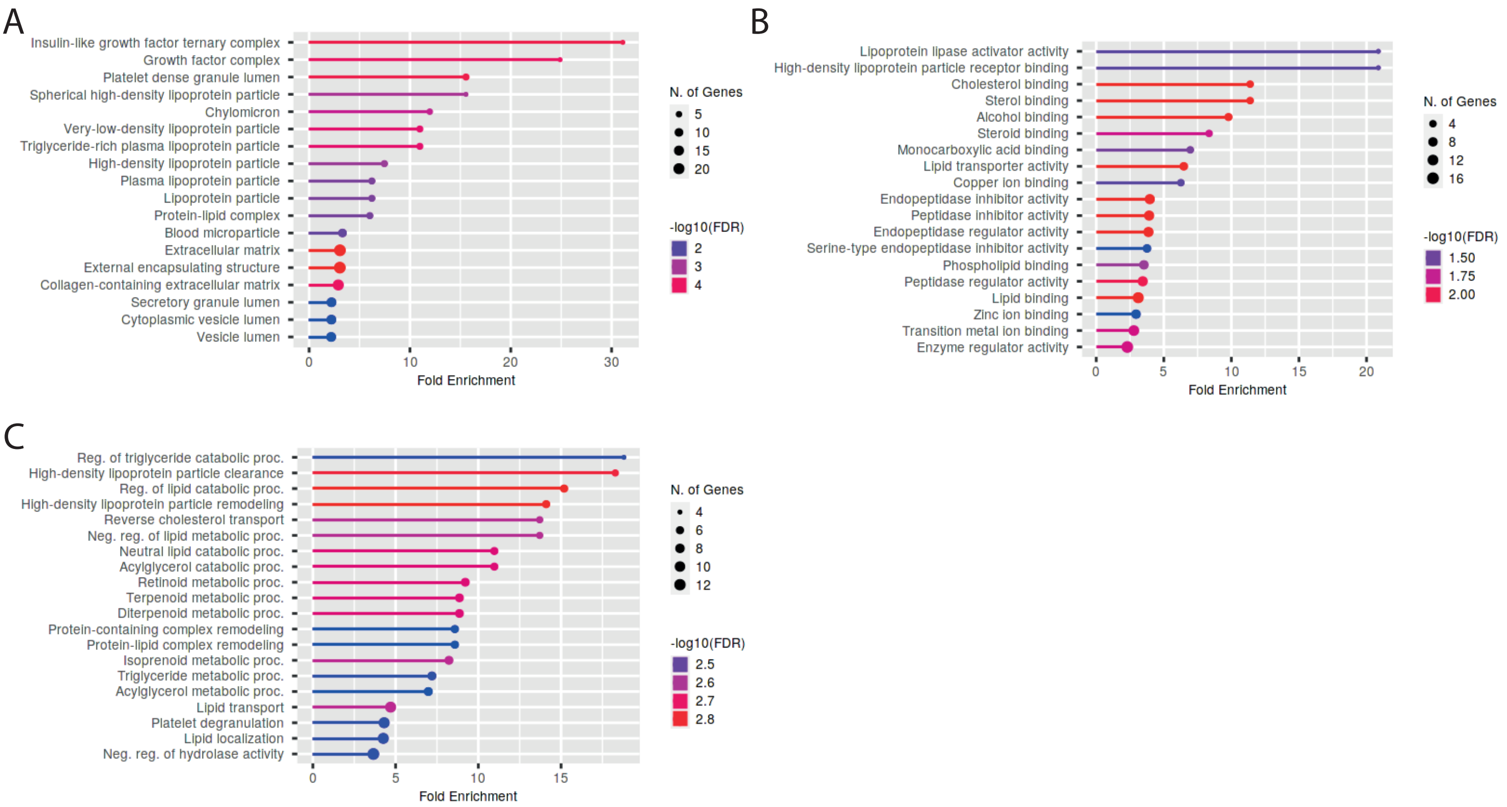

S5: Gene ontology analysis of significantly downregulated proteins.
Lollipop plots displaying fold enrichment and significance as false discovery rate (FDR) of ontology terms for (A) cellular compartment (B) molecular function (C) biological processes of downregulated proteins. The length of the lollipop is the fold enrichment of the pathway, the size of lollipop head indicates the number of proteins in the input dataset that are found within the pathway and the colour indicates the statistical significance of the enrichment. Gene ontology enrichment performed using ShinyGO against the background of the entire plasma proteome identified from discovery mass spectrometry proteomics.

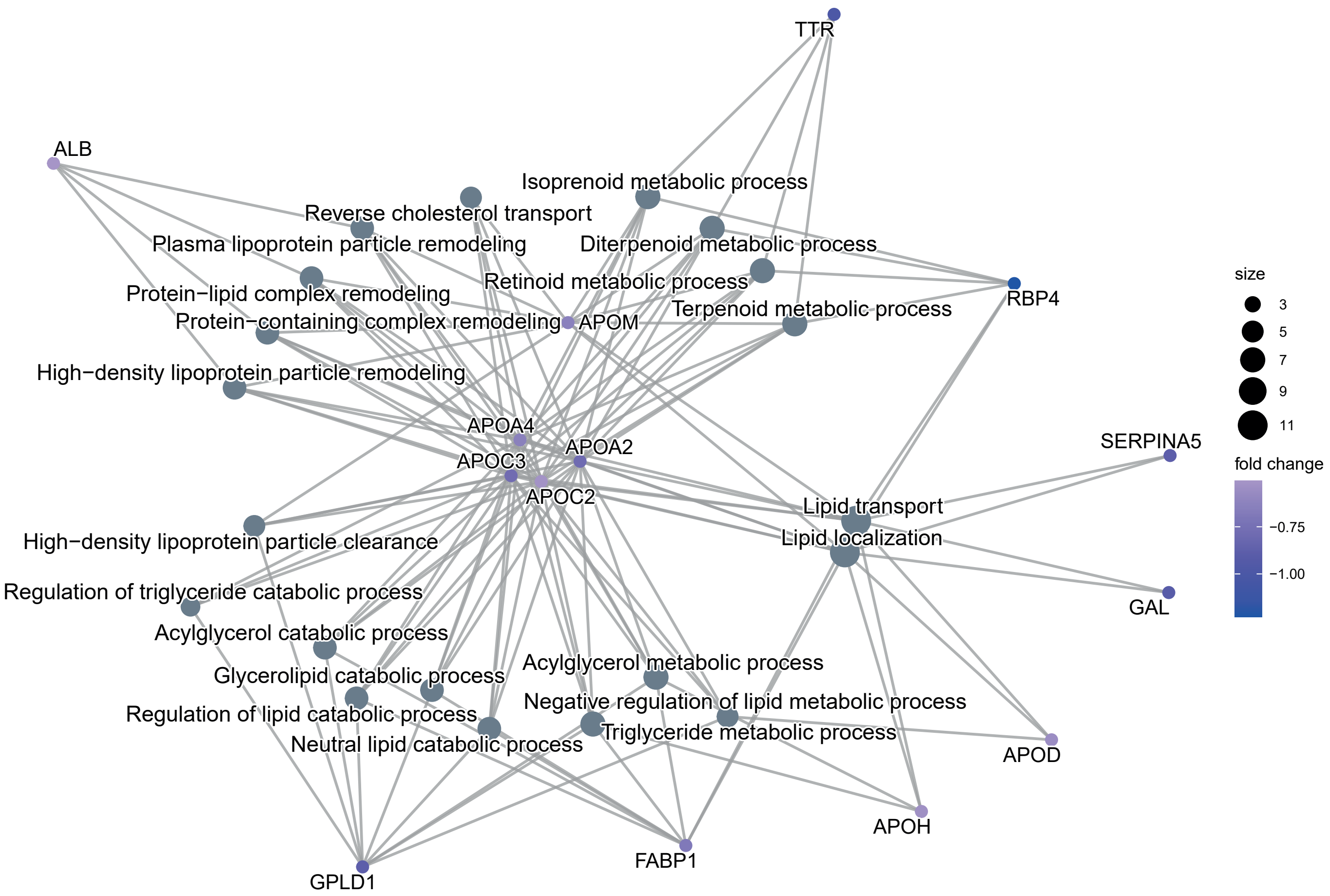

S6: Concept network plot of significantly downregulated proteins and their enriched biological processes.
Plot generated from ShinyGO enrichment by biological process of downregulated proteins using the cnetplot function in the R package GOplots. The top 20 most enriched pathways are displayed linked to their relevant differentially expressed proteins.

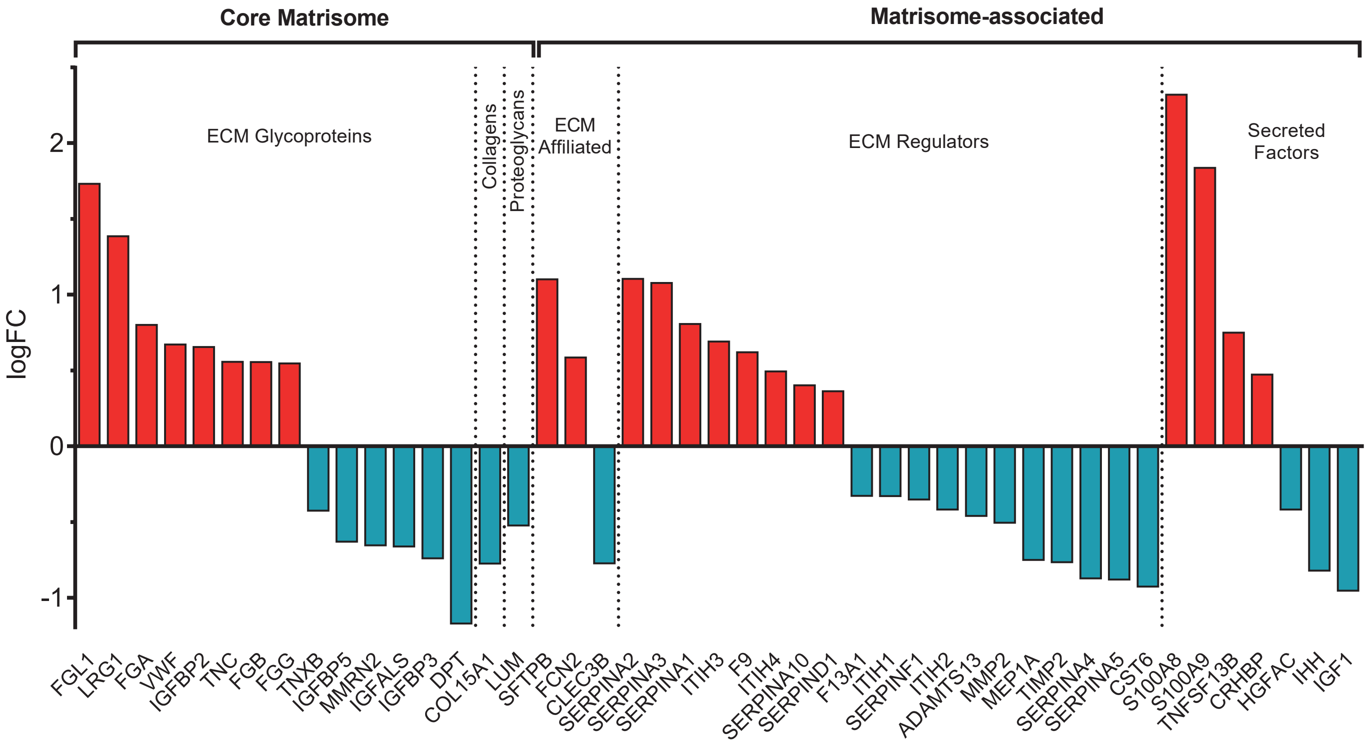

S7: Differential expression of 'matrisome'-associated proteins in the plasma of active pulmonary TB patients.
45 of 118 (38%) of differentially expressed plasma proteins in active pulmonary TB are contained within the 'matrisome', an ensemble of ~300 genes which encode the core extracellular matrix (ECM) and a further ~700 genes which encode ECM associated and regulatory proteins. Matrisome data accessed from http://matrisomeproject.mit.edu/other-resources/human-matrisome/ in September 2022.

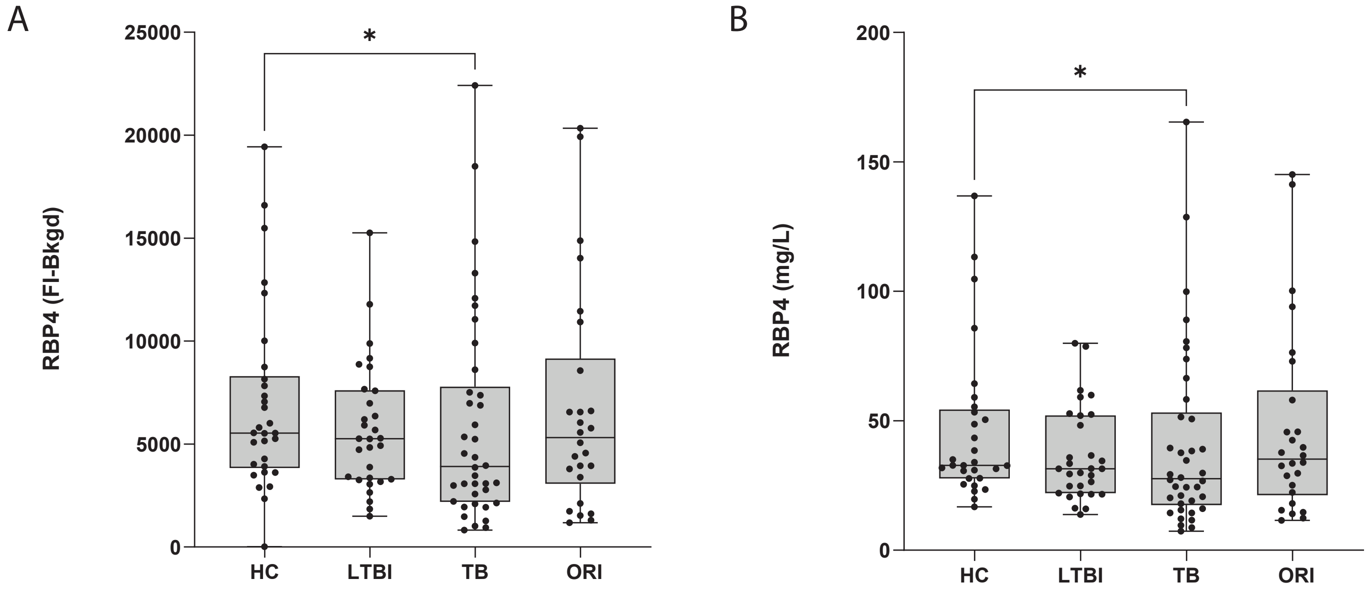

S8: RBP4 is significantly downregulated in the plasma of patients with active pulmonary TB

(A) Box and whisker plot of fluorescence intensity values minus background levels in contrasting clinical groups of the MIMIC cohort. (B) Box and whisker plot of RBP4 serum concentration showing significant downregulation of RBP4 in active pulmonary TB. Values measured by Luminex assay.

HC healthy control; LTBI latent TB infection; TB active pulmonary TB; ORI other respiratory infections; * $p \leq 0.05$

A

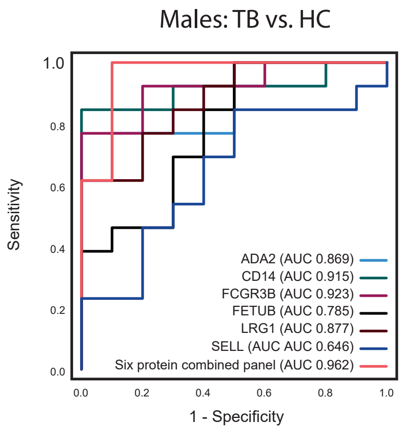

B

	Predicted			
Observed	HC	TB	% correct	
HC	9	1	90.0	Specificity
TB	1	12	92.3	Sensitivity
Overall %			91.3	Accuracy

C

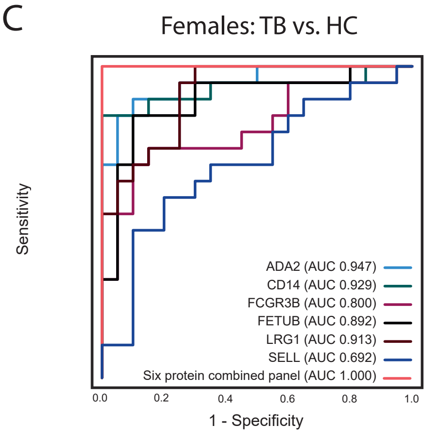

D

	Predicted			
Observed	HC	TB	% correct	
HC	20	0	100.0	Specificity
TB	0	19	100.0	Sensitivity
Overall %			100.0	Accuracy

E

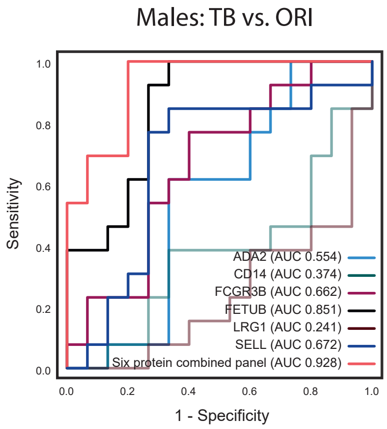

F

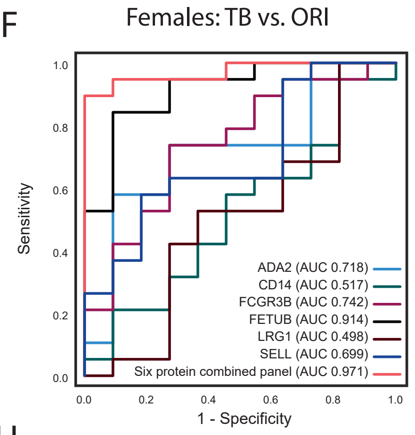

G

	Predicted			
Observed	ORI	TB	% correct	
ORI	12	3	80.0	Specificity
TB	1	12	92.3	Sensitivity
Overall %			85.7	Accuracy

H

	Predicted			
Observed	ORI	TB	% correct	
ORI	8	3	72.7	Specificity
TB	1	18	94.7	Sensitivity
Overall %			86.7	Accuracy

S9: Diagnostic performance of the final six protein panel in the UK MIMIC Cohort disaggregated by sex

(A) ROC curve and (B) classification grid of the final six protein panel demonstrating discrimination of male patients with TB from male healthy controls (AUC 0.962, sensitivity 92.3%, specificity 90.9%)

(C) ROC curve and (D) classification grid of the final six protein panel demonstrating discrimination of female patients with TB from female healthy controls (AUC 1.000, sensitivity 100%, specificity 100%)

(E) ROC curve and (F) classification grid of the final six protein panel demonstrating discrimination of male patients with TB from male ORI (AUC 0.928, sensitivity 92.3%, specificity 80.0%)

(G) ROC curve and (H) classification grid of the final six protein panel demonstrating discrimination of female patients with TB from female ORI (AUC 0.971, sensitivity 94.7%, specificity 72.7%)

All ROC curves and classification grids were generated using SPSS v28.0.1.0 after binary logistic regression for combined proteins. AUC were calculated under non-parametric assumption. TB was set as the positive test outcome and the test direction such that a larger test result indicates a more positive test.

ADA2: adenosine deaminase 2; CD14: monocyte differentiation antigen; FCGR3B: low-affinity immunoglobulin receptor 3B; FETUB: fetuin-B; LRG1: leucine-rich alpha-2-glycoprotein; SELL: L-selectin. TB: tuberculosis; HC: healthy control; ORI: other respiratory infection

A

Cohort 1: TB vs. both healthy controls & other respiratory infection

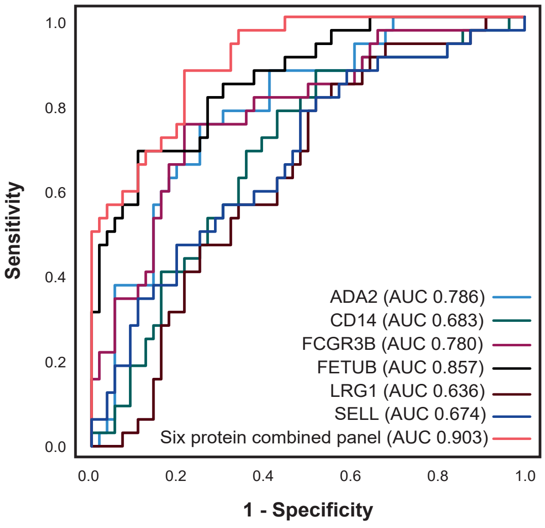

C

Cohort 2: TB vs. both healthy controls & other respiratory infection

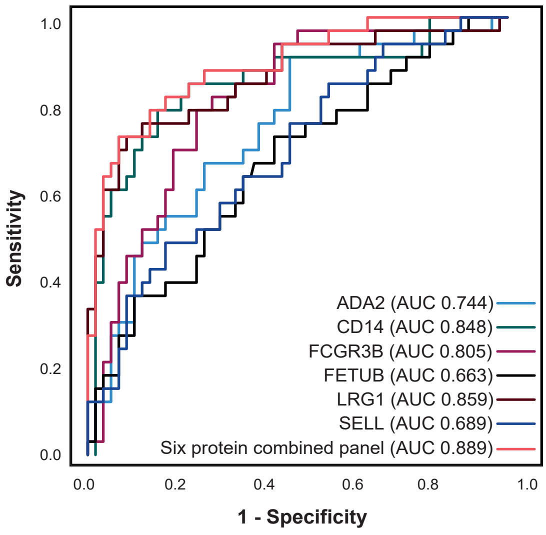

B

Observed	Predicted HC & ORI	Predicted TB	% correct	
HC & ORI	50	6	89.3	Specificity
TB	12	20	62.5	Sensitivity
Overall %			79.5	Accuracy

D

Observed	Predicted HC & ORI	Predicted TB	% correct	
HC & ORI	49	5	90.7	Specificity
TB	9	24	72.7	Sensitivity
Overall %			83.9	Accuracy

S10: The final combined six protein panel discriminates patients with TB from a combined group of both healthy controls and other respiratory infections with high specificity in both patient cohorts

(A) ROC curve and (B) classification grid of the final six protein panel comprising FCGR3B, FETUB, LRG1, ADA2, CD14 and SELL, demonstrating discrimination of patients with TB from both healthy controls and other respiratory infection as a combined group in Cohort 1 (AUC 0.903, sensitivity 62.5%, specificity 89.3%)

(C) ROC curve and (D) classification grid of the final six protein panel comprising FCGR3B, FETUB, LRG1, ADA2, CD14 and SELL, demonstrating discrimination of patients with TB from both healthy controls and other respiratory infection as a combined group in Cohort 2 (AUC 0.889, sensitivity 72.7%, specificity 90.7%).

ROC curves and classification grids were generated using SPSS v28.0.1.0 after binary logistic regression for combined proteins. AUC was calculated under non-parametric assumption. TB was set as the positive test outcome and the test direction such that a larger test result indicates a more positive test.

ADA2: adenosine deaminase 2; CD14: monocyte differentiation antigen; FCGR3B: low-affinity immunoglobulin receptor 3B; FETUB: fetuin-B; LRG1: leucine-rich alpha-2-glycoprotein; SELL: L-selectin. TB: tuberculosis; HC: healthy control; ORI: other respiratory infection
